# Mathematical Modeling of the Dynamics of Shoot-Root Interactions and Resource Partitioning in Plant Growth

**DOI:** 10.1371/journal.pone.0127905

**Published:** 2015-07-08

**Authors:** Chrystel Feller, Patrick Favre, Ales Janka, Samuel C. Zeeman, Jean-Pierre Gabriel, Didier Reinhardt

**Affiliations:** 1 Dept. of Mathematics, University of Fribourg, Fribourg, Switzerland; 2 Dept. of Biology, University of Fribourg, Fribourg, Switzerland; 3 Institute of Agricultural Sciences, ETH Zürich, Zürich, Switzerland; University of Nottingham, UNITED KINGDOM

## Abstract

Plants are highly plastic in their potential to adapt to changing environmental conditions. For example, they can selectively promote the relative growth of the root and the shoot in response to limiting supply of mineral nutrients and light, respectively, a phenomenon that is referred to as balanced growth or functional equilibrium. To gain insight into the regulatory network that controls this phenomenon, we took a systems biology approach that combines experimental work with mathematical modeling. We developed a mathematical model representing the activities of the root (nutrient and water uptake) and the shoot (photosynthesis), and their interactions through the exchange of the substrates sugar and phosphate (P_i_). The model has been calibrated and validated with two independent experimental data sets obtained with *Petunia hybrida*. It involves a realistic environment with a day-and-night cycle, which necessitated the introduction of a transitory carbohydrate storage pool and an endogenous clock for coordination of metabolism with the environment. Our main goal was to grasp the dynamic adaptation of shoot:root ratio as a result of changes in light and P_i_ supply. The results of our study are in agreement with balanced growth hypothesis, suggesting that plants maintain a functional equilibrium between shoot and root activity based on differential growth of these two compartments. Furthermore, our results indicate that resource partitioning can be understood as the emergent property of many local physiological processes in the shoot and the root without explicit partitioning functions. Based on its encouraging predictive power, the model will be further developed as a tool to analyze resource partitioning in shoot and root crops.

## Introduction

For optimal development of the plant as a whole, root and shoot biomass have to be balanced. In a given environment, the root fraction (RF; the ratio of root mass relative to the mass of the entire plant) lies within certain species-specific limits, suggesting that the relative growth of the root and the shoot has a genetic basis. Under varying environmental conditions, however, the relative growth of the shoot and the root can change. For example, when light is limiting, the RF can change in favor of the shoot [1–3]. Conversely, root fraction increases when the supply of mineral nutrients such as inorganic P_i_ becomes limiting [4, 5]. These adaptive growth responses suggest that plants possess mechanisms for the control of the relative partitioning of their resources to the shoot and the root [6]. It is generally assumed that the organ providing the limiting resource is prioritized [5], a concept that has been termed”balanced growth hypothesis” [7–9], or "functional equilibrium" [10]. Although this phenomenon has been described in various species, the underlying molecular and physiological mechanisms remain elusive.

A plausible control mechanism for organ growth is the regulation of relative assimilate allocation [11, 12]. In addition, sink organs can potentially stimulate sugar supply by activating their consumption rate, thereby increasing their sink strength [13]. Consequently, relative carbon allocation to a particular organ must be regarded as a function of source and sink activities of all parts of the plant [14], and therefore, a better understanding of local aspects of partitioning requires a global view of resource allocation. In addition to the intricate spatial organization of the resource fluxes in the plant, the interactions among the different plant parts adapt dynamically to changes in environmental factors such as light and nutrient supply, implying complex mechanisms in the spatio-temporal regulation of resource partitioning.

To reach an integrated view of resource partitioning, information from physiological studies has been introduced into mathematical models of partitioning (reviewed in [15]). Models of carbon allocation can be roughly divided into empirical, teleonomic and mechanistic models [10, 16]. Empirical models can reproduce the observed behaviour of plants, but are not necessarily based on biological principles. In teleonomic models, the relative allocation of resources to the root and the shoot is assumed to be distributed according to a central partitioning function [8, 17, 18]. Some teleonomic models explicitly involve a concept of maximization (e.g. [19–21]), assuming that plants can extrapolate their growth behavior and opt for a strategy that results in maximal overall growth in the long run. Although teleonomic models are usually simple and some can closely mimic plant behavior, they lack support from physiological evidence, since potential goal-seeking mechanisms in plants are not known and it is not clear where in the plant a central partitioning function would operate.

Mechanistic models are based on detailed physiological knowledge derived from experimentation [10, 16, 22]. Importantly, they do not invoke central regulatory mechanisms as in teleonomic models, but are based exclusively on local physiological processes in the plant. A realistic model would be entirely mechanistic, however, our understanding of several processes related to plant growth are fragmentary. Consequently, empirical concepts are needed to incorporate simplified mechanistic principles into integrated growth models. Based on the assumption that transport of resources between source and sink tissues may represent a limiting factor in resource partitioning, several mechanistic growth models describe differential growth phenomena as a function of transport resistance [22–24].

A plausible mechanistic model of plant growth has been proposed by Thornley [24–26]. Its central tenet is that relative growth of the roots and the shoot results from the relative allocation of carbon between these organs as the outcome of substrate supply, transport, and utilization. With its original parameter set, this model reproduced several aspects of adaptive plant behavior in response to the environment [15] such as an increase of root fraction under conditions of P_i_ (or nitrogen) starvation, or a decrease of root fraction as a result of reduced photosynthesis rate. Taking a similar approach, we set out to construct a mechanistic model to study root-shoot interactions in a realistic context. However, in contrast to previous partitioning models that often involve asymptotic convergence towards a point of equilibrium, our model is intended to grasp the transitory behavior during adaptive growth with realistic dynamics.

Here, we describe a partitioning model of *Petunia hybrida* that is embedded in a realistic environment with a day-and-night cycle. The model involves carbohydrate reserves (starch) for the dark period, and takes into account the complex regulation involved in the diurnal switch between photosynthetic and heterotrophic metabolism, because these processes are likely to impact on global resource allocation and plant growth in the long run. Sugar transport from source to sink is modeled as mass flow driven by osmotic pressure. An internal oscillator was introduced as a circadian clock to coordinate plant metabolism with the environment. We also took into account metabolic costs for respiration, nutrient uptake, transport and growth. In addition, we consider that light intensity influences leaf thickness [2, 6, 27–29], thereby impacting on relative photosynthetically active leaf area. Finally, in agreement with the notion of Marcelis et al. [30] that ‘more attention should be paid to validation of the models under a wide range of conditions using independent data sets’, our model has been calibrated and validated with two independent experimental data sets. Our results show that both, the diurnal changes in resource allocation associated with the day-and-night cycle, as well as the slower changes resulting from modified nutrient and light supply, can be explained as the emergent outcome of all the local events in the different plant parts, and their direct and indirect interactions, without an explicit partitioning function. This model will be further developed and adapted to shoot and root crops in order to address the dynamics of their resource allocation under various conditions. Such mechanistic models will be valuable tools to aid interpretation of the complex phenotypes of mutants affected in carbohydrate metabolism and the circadian clock, and they will help design new strategies in molecular breeding for the improvement of crop performance.

## Material and Methods

### Experimental procedures

#### Plant growth conditions

*Petunia hybrida* W115 (cv Mitchell) seedlings were grown in a thermo- and hygro-regulated growth chamber (22 ± 2 °C, 40–60% rH) under 12 h: 12 h light and dark (L: D, Sylvania 36W Luxline-Plus, 250 μmol m^−2^ s^−1^ photosynthetically active radiation). They were first germinated and grown for two weeks in containers with seedling substrate (Klasmann-Dilmann GmbH, Germany) covered with transparent plastic lids. Subsequently, the seedlings were transferred and grown individually for two additional weeks in a substrate consisting of 70% sand and 30% unfertilized loamy soil with low nutrient content [31]. Plants grew in ca. 175 ml substrate per plant in 40-well trays (Eric Schweizer SA, Switzerland), and were watered twice weekly with nutrient solution (Planta-aktiv 18+0+22 typ NK, Hauert HBG Dünger AG, Switzerland) supplemented with 200 μM KH_2_PO_4_. Then, the plants were transferred to 300 ml pots containing quartz sand (0.1–0.7 mm grain size, Carlo Bernasconi SA, Switzerland). They were supplied with fertilizer solution (3mM MgSO_4_, 0.75mM KNO_3_, 0.87 mM KCl, 1.52 mM Ca(NO_3_)_2_, 20 μM NaFeIII EDTA, 11 μM MnSO_4_, 1 μM ZnSO_4_, 30 μM H_3_BO_3_, 0.96 μM CuSO_4_, 0.03 μM (NH_4_)_6_Mo_7_O24, and 0.01 μM Na_2_MoO_4_) containing various concentrations of KH_2_PO_4_ from 10 μM to 1 mM as indicated and grown at different photon flux densities from 90 to 600 μmol m^−2^ s^−1^ PAR (photosynthetically active radiation) (12 h: 12 h light: dark). In order to maintain nutrient concentrations in the soil approximately constant during the experiment, plants were watered three times per week with 200 ml of the respective nutrient solutions. This represents an approximate four-fold excess volume of solution and led to extensive leaching, hence, the actual solution in the sand substrate was replaced, leading to a reset to the original nutrient concentrations. Thus, instead of controlling the amount of nutrients delivered to each plant, we aimed at maintaining nearly constant nutrient concentrations during the experiment. Plants remained in their vegetative rosette stage throughout the entire range of the experiments.

#### Analysis of plant architecture

To evaluate the photosynthetically active leaf surface, plants were grown under high, medium and low light intensity (450, 191, and 93 μmol m^-2^s^-1^, respectively) and watered with a high level of P_i_ supply (300 μM KH_2_PO_4_). Shoots of plants were weighed and projected surface area was captured with a camera (Nikon, coolpix S4). Leaf thickness was measured on images of transverse sections of mature leaves with a binocular microscope (Leica MZFLIII, Nikon digital sight DS-U1). To estimate phloem tube length, the length of the leaves and of the root system was measured from plants with high light and P_i_ supply at an age of 43, 51, 57, 64, and 69 days after sowing. Tube length was defined as the average distance between the centres af the leaf blade of the fully expanded rosette leaves (source) and the root tips (sink). The number and diameter of phloem tubes were estimated by callose staining (reveals sieve plates) and confocal microscopy. Briefly, transverse hand sections of hypocotyls from plants at an age of 43, 51, 57, 64, and 69 days were fixed by immersion in 6:1 ethanol: lactic acid (vol: vol), followed by progressive rehydration in 70% ethanol, 50% ethanol for 2 hours each, and overnight incubation in water. Samples were then incubated for 24 hours in 150 mM KH_2_PO_4_ (pH 9.5) containing 0.01% aniline blue. Subsequently, sections were counterstained with 0.01% propidium iodide and mounted in 50% glycerol for analysis by laser confocal scanning microscopy (Leica TCS SP5). The number and diameter of phloem tubes were determined with ImageJ 1.43 (NIH) on sections by automatic quantification of pixels in the green channel whithin the zone that comprises the phloem region (see Supporting Information).

#### Determination of plant phosphate content

The amount of soluble P_i_ in samples was determined according to [32] with the following modifications. Fresh tissue was sampled (0.2–0.3 g) and macerated in 4 ml extract buffer (45 mM sodium acetate buffer, pH 5.0). The extract was then centrifuged at 10’300 g for 10 min. Soluble P_i_ was determined with the P_i_ reagent (1% ammonium heptamolybdate, 0.5% ammonium metavanadate, 14% nitric acid) by measuring light absorbance at 405 nm (absorbance from pigment in the plant extract was subtracted with a double blank). For the determination of insoluble P_i_, the samples were then washed with distilled water, dried and ashed overnight at 550° C. Ash was dissolved in 2 ml HCl (0.5 M), and centrifuged at 10’300 g for 10 min. P_i_ content was determined as described above. Total P_i_ content represents the sum of soluble and insoluble P_i_ content.

#### Sugar determination

Sugars of fresh leaves and roots were extracted as described in [33]. Sucrose, glucose and fructose were measured spectrophotometrically in an assay cocktail containing glucose 6-phosphate dehydrogenase, by adding sequentially hexokinase, phosphoglucose isomerase and invertase according to [34, 35]. Soluble sugar represents the sum of sucrose, glucose and fructose.

#### Determinations of physical parameters

In order to calculate shoot and root FW/DW ratio, fresh samples were weighed, then dried at 80° C and weighed again. Density of fresh tissue was obtained by measuring FW of leaves and root, and their corresponding volume in 0.1% (v/v) Triton X-405 (Fluka) that allows the interconversion of cm^3^ and g.

#### Experimental design and plant treatments

Experiment 1: To determine the maximal growth rate, plants were grown under favorable conditions i.e. with a saturating light intensity of 595 μmol m^-2^s^-1^ and with a P_i_ supply of 300 μM KH_2_PO_4_.

**Fig 1:**
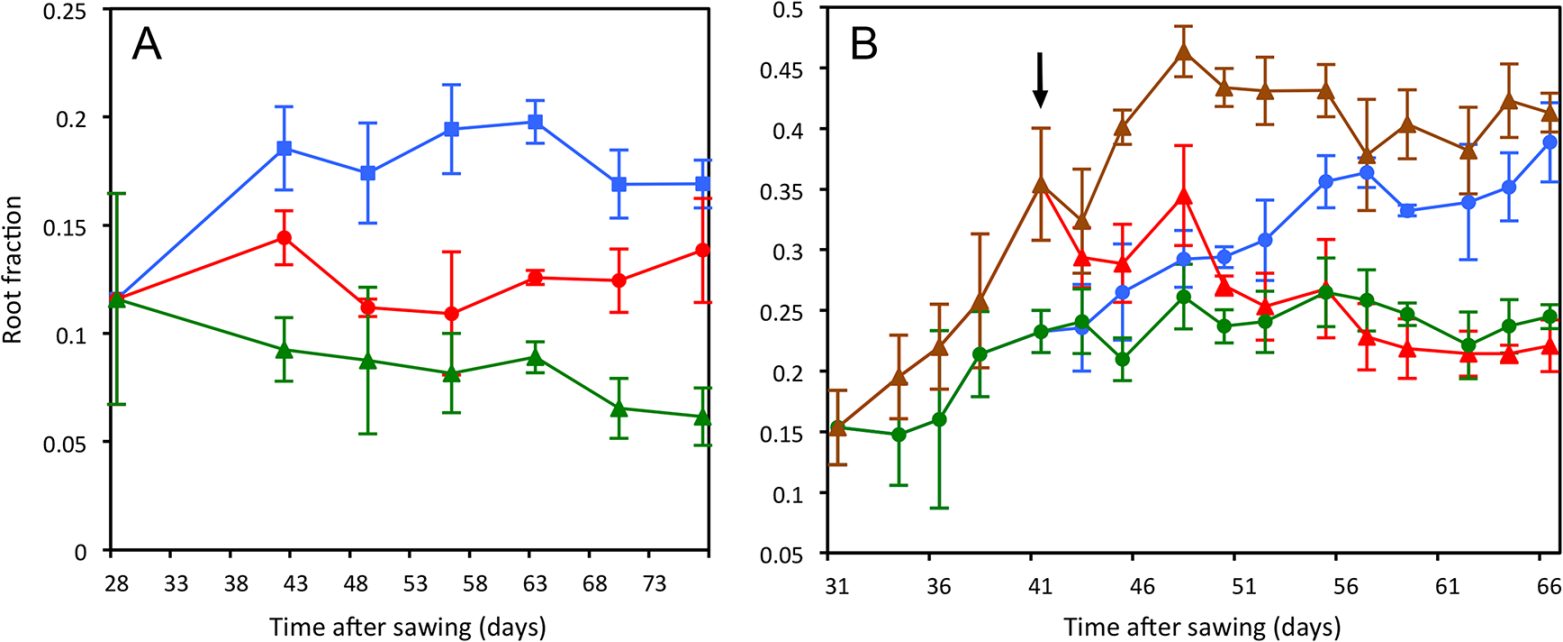
Adaptive response of *P*. *hybrida* to different light and P_i_ supply. (A) Effects of light intensity on root fraction. Plants were grown under 450 μmol m^-2^ s^-1^ (circles), 191 μmol m^-2^ s^-1^ (squares) or 93 μmol m^-2^ s^-1^ (triangles). Root fraction was determined between 28 and 78 days after germination. Error bars represent the standard deviations (N = 3). (B) Effects of P_i_ supply on root fraction in *P*. *hybrida*. Plants were grown with 10 μM KH_2_PO_4_ (triangles) or with 100 μM KH_2_PO_4_ (circles). In two additional treatments, plants were transferred from low to high (open triangles), or from high to low P_i_ supply (open circles) at 41 days after sowing (arrow). Root fraction is defined as the proportion of root fresh weight divided by the fresh weight of the entire plant. Error bars represent the standard deviations (N = 5).

Experiment 2: To explore the adaptive potential of petunia to P_i_ supply, plants were grown under a light intensity of 316 μmol m^-2^s^-1^ and with a P_i_ (KH_2_PO_4_) supply of 10 μM (experiment 2, treatment A) and 100 μM (experiment 2, treatment B), respectively. In two additional treatments, the nutrient solutions were swapped after 43 days: From 10 μM to 100 μM (experiment 2, treatment C) and from 100 μM to 10 μM KH_2_PO_4_ (experiment 2, treatment D; experimental design as in Fig 1).

Experiment 3: To further evaluate the adaptive potential of plants to P_i_ supply, plants were grown under high light (372 μmol m^-2^s^-1^) and a range of different P_i_ concentrations in the soil (1, 10, 30, 100, 300, 1000 μM).

### Characterization of the model

The mathematical model for plant growth is inspired by several previous models in particular of Thornley and Dewar [24, 25, 36], and incorporates additional features that are essential for a realistic understanding of plant growth under natural conditions. The model consists of mechanistic principles and simplified empirical elements as described below and in the Supporting Information. In order to give a complete overview of the components of our mathematical model, we provide here a detailed description of the assumptions and hypotheses on which the model is based.

#### General assumptions and hypotheses

In short, the model is characterized by the following assumptions and hypotheses that are described in detail in the following paragraphs (see also Supporting Information).

**Fig 2:**
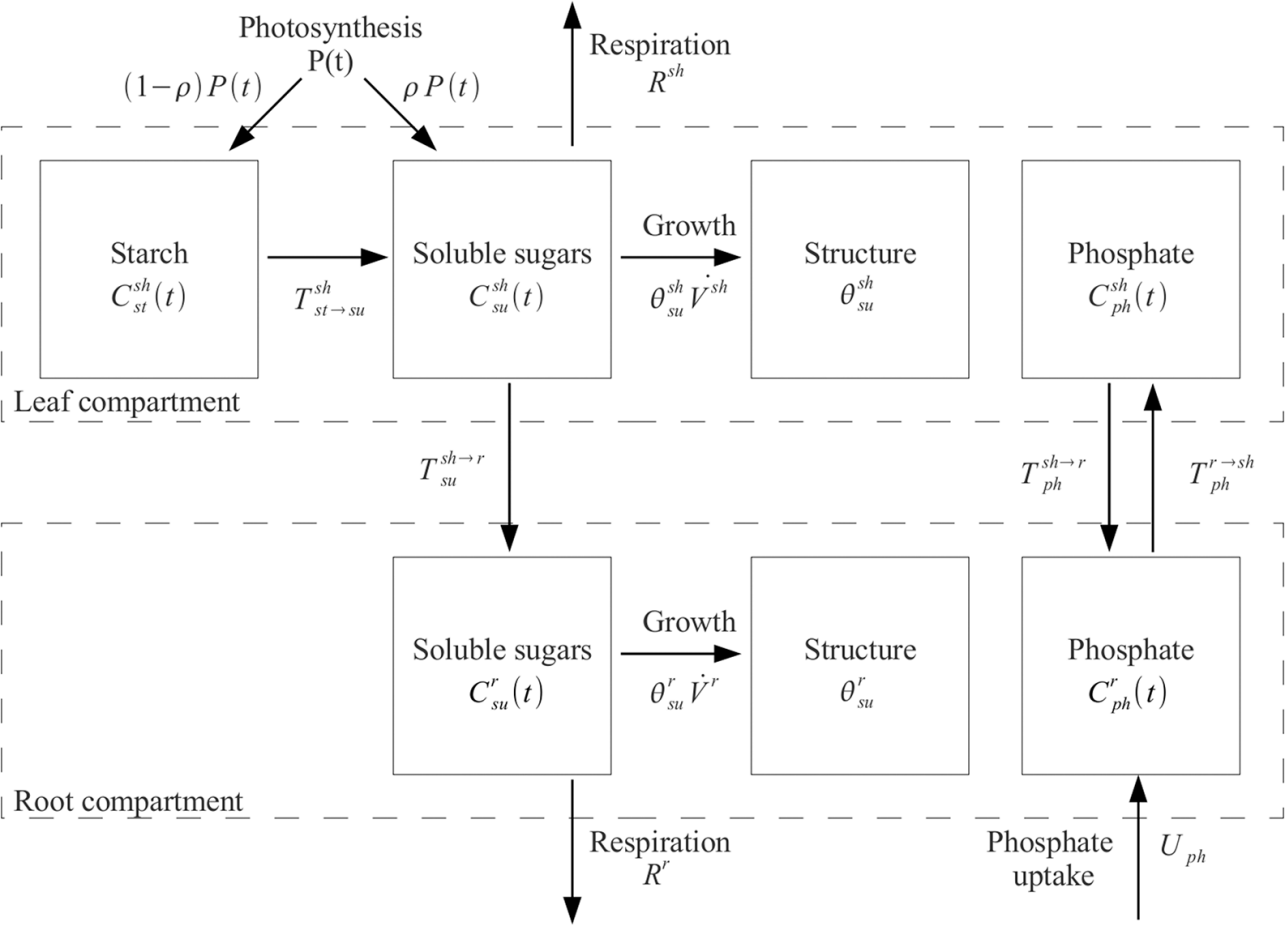
Schematic representation of the plant model and its architecture. Arrows represent the flux of carbon (sugar, starch or CO_2_) and P_i_ between the shoot and the root compartments. Sugar produced by photosynthesis is either stored (starch) or released to the soluble pool where it becomes available for growth, respiration, or transport to the root. P_i_ is transported from the soil to the soluble pool of the root compartment and transferred to the shoot. A fraction of the P_i_ is transported back to the root compartment together with sugar flux in the phloem.

- The model involves two compartments, the shoot and the root (Fig 2);
- Both compartments are subdivided into a solid part representing structural components (corresponding to cell walls) and a pool containing the soluble substrates (corresponding to the cytoplasm);
- The shoot contains another sub-compartment for storage of C as starch (Fig 2);
- Reduced carbon (C) occurs in a soluble form (sugar) and an insoluble form (starch).
- P_i_ serves as a representative for all mineral nutrients; other nutrients are not considered here;
- P_i_ concentration in the soil is re-initialized thrice weekly (as in the experiments).
- Plants are growing under a light intensity *I*(*t*) oscillating between 0 (during the night) and the actual light intensity during the day *J*, i.e.:

I(t)=J⋅D(t)

where *D*(*t*) is a continuous function equal to 0 during the night and 1 during the day with a transition of 30 min in between;

- The plant is not limited by water supply. The quantity of water per cm^3^ of plant is constant, denoted by $d_{H_{2}O}^{p}$;
- The state of the system plant-soil is described with 9 time-dependent variables (Table 1).

**Table 1:** Plant state variables.

No	Variable	Definition	Unit
1	$Q_{ph}^{soil}(t)$	soluble P_i_ quantity in the soil at time *t*	μg
2	$Q_{su}^{sh}(t)$	soluble sugar quantity in the shoot compartment at time *t*	μg
3	$Q_{su}^{r}(t)$	soluble sugar quantity in the root compartment at time *t*	μg
4	$Q_{st}^{sh}(t)$	starch quantity in the storage pool of the shoot compartment at time *t*	μg
5	*ρ*(*t*)	fraction of photosynthates that is allocated to the soluble pool at time *t*	μg μg^−1^
6	$Q_{ph}^{sh}(t)$	P_i_ quantity in the shoot compartment at time *t*	μg
7	$Q_{ph}^{r}(t)$	P_i_ quantity in the root compartment at time *t*	μg
8	*V* ^*sh*^(*t*)	shoot volume at time *t*	cm^3^
9	*V* ^*r*^ (*t*)	root volume at time *t*	cm^3^
	→	The actual concentrations at any time are defined in relation to the respective volumes.	

The following three types of function were used to introduce feedback regulation and saturation kinetics (Fig A in S1 File):

1. The Monod function *M*(*x*) (Fig A in S1 File, panel a) defined by
  $$M\left(x\right)=\frac{x}{m+x}\;\text{for}\;x\geq 0$$
  where *m* is a positive parameter, with the property that $M\left(m\right)=\frac{1}{2}$.
2. Positive regulatory function with saturation: *S* ^+^ (*x*) (Fig A in S1 File, panel b) defined by
  $$S^{+}\left(x\right)=\frac{{\left(x-u\right)}^{2}}{a+{\left(x-u\right)}^{2}}\text{if}\;x\geq u\;\text{and}\;S^{+}\left(x\right)=0\;\text{otherwise}$$
  where *a* > 0 and *u* ≥ 0 are parameters.
3. Negative regulatory asymptotic function with *S* ^−^ (*x*) (Fig A in S1 File, panel c) given by
  $$S^{-}\left(x\right)=s_{d}+\left(1-s_{d}\right)\frac{{\left(u-x\right)}^{2}}{s+{\left(u-x\right)}^{2}}\frac{s+u^{2}}{u^{2}}\;\text{if}\;x\leq u\;\text{and}\;S^{-}\left(x\right)=s_{d}\;\text{otherwise}$$
  where *s* > 0, *s*_*d*_ ≥ 0 and *u* > 0 are parameters. Clearly *S* ^−^ (0) = 1. For *u* = 0, we define:
  $$S^{-}\left(x\right)=s_{d}+\left(1-s_{d}\right)\frac{s}{s+x^{2}}\;\text{if}\;x\geq 0.$$

#### Hypotheses and assumptions concerning plant architecture

Empirical submodels were developed for the photosynthetically active surface area, and the number and length of phloem tubes. These empirical submodels were tested on datasets and the related parameters were estimated in the Supporting Information (see Figs B-D in S1 File).

In general, the following notation was used for derivatives in time $\dot{X=\frac{dX}{dt}}$.

#### Hypothesis 1 (Leaf architecture)

Leaf thickness is correlated with light intensity. This means that plants can adapt to weak light conditions by increasing leaf surface at the expense of leaf thickness, thereby increasing the relative absorption of the limiting resource [2, 6, 27–29]. Hence, leaf thickness *th* (in cm) depends on light intensity *J* through the following relation:

$$th\left(J\right)=th_{min}+\left(th_{max}-th_{min}\right)M_{th}\left(J\right)$$

where *th*_*min*_ and *th*_*max*_ are the minimal and the maximal leaf thickness (in cm) and *M*_*th*_a Monod function with parameter *m*_*th*_ (Fig A in S1 File, panel a). *J* is the respective constant light intensity at a given setting and varies between experiments and simulations as indicated in the text.

#### Hypothesis 2 (Photosynthetically active leaf surface)

In young plants with few leaves, mutual shading of the leaves is negligible, however, at later stages, new leaves shade the older ones [37]. To take account of this fact, we approximate the photoactive leaf surface $S_{photo}^{sh}$ by $\frac{V^{sh}}{th\left(J\right)}$. With increasing leaf number, mutual shading of leaves progressively increases and from a critical surface *S*_*c*_ onward, $S_{photo}^{sh}$ becomes sublinear. For a rosette plant, we take the surface of a hemisphere as an approximation for the final photosynthetically active surface area. Therefore:

$$S_{photo}^{sh}=\{\begin{array}{ll} \frac{V^{sh}(t)}{th(I_{max})} & \;if\;V^{sh}(t)\leq S_{c}\cdot th(I_{max})\\ \lambda_{1}\cdot {\left(V^{sh}(t)\right)}^{\frac{2}{3}}+\lambda_{2} & \;otherwise \end{array}$$

Where $\lambda_{1}=\frac{3}{2}S_{c}^{1/3}th{\left(I_{max}\right)}^{-2/3}$, and $\lambda_{2}=-\frac{1}{2}S_{c}$ were chosen such that $S_{photo}^{sh}$ is a continuously differentiable function of *V* ^*sh*^. Our first attempts for the photosynthetically active leaf surface were based on $\frac{V^{sh}}{th}$ where *th* was assumed to be a function of the light intensity, the shoot volume, or both, or none of them. These attempts were unsatisfactory, and were therefore dismissed. The best model turned out to be the one described above, which resulted in a good fit with a small number of parameters (see Fig B in S1 File). The superior quality of this latter submodel was confirmed by comparing all versions based on their weighed sum of the quadratic errors between simulated and experimental values (data not shown).

#### Hypothesis 3 (Root surface active in nutrient uptake)

P_i_ uptake is limited to young roots. In an aged plant this involves essentially the outer surface of the soil volume that has already been explored by the plant. This volume represents a P_i_ depletion zone, hence P_i_ can only be acquired at the outer root front of this depletion zone [38]. In an annual plant like petunia, the root system tends to a maximal expansion during the life cycle of the plant. To account for this behavior, the root surface active in P_i_ uptake in the model is initially proportional to the root volume (in the young plant), and later tends towards a constant *S*_*max*_ with the root volume:

$$S_{active}^{r}=S_{max}M_{as}\left(V^{r}\right)$$

The parameter of *M*_*as*_ is denoted *m*_*as*_. This reflects the fact that only young roots can absorb P_i_, whereas an increasing proportion of the growing root system is inactive in P_i_ uptake.

#### Hypothesis 4 (Phloem tube length)

The phloem connects the photosynthetic tissues in the leaves with the heterotrophic (energy-consuming) tissues of the root meristems [39, 40]. Hence, the length of the phloem tubes in the model corresponds to the average distance from the photosynthetic source leaves (represented by their centres) to the root tips. Phloem tube length L increases as a function of plant volume *V* ^*pl*^ (*t*) given by

$$L\left(V^{pl}\left(t\right)\right)=l_{1}M_{L}\left(V^{pl}\left(t\right)\right)+l_{2}V^{pl}\left(t\right)$$

where $l_{1},l_{2}$ are positive parameters. The parameter of *M*_*L*_ is denoted with *m*_*L*_.

In an earlier version, phloem tube length was modelled with a Monod function only, but this resulted in an unsatisfactory fit and was therefore extended with a linear component (Fig C in S1 File).

#### Hypothesis 5 (Number of phloem tubes)

Phloem tubes occur as separate strands in young stems and merge to concentric rings around the woody tissues after the onset of secondary growth [41]. Individual phloem strands are thin longitudinal files of cells that can be identified based on their callose content [42]. We have used this feature to estimate the number of phloem tubes in petunia at different developmental stages (Fig D in S1 File). In the model, the number of phloem tubes *n* is an increasing function of plant volume *V* ^*pl*^ (*t*). In young plants, the function appeared exponential (Fig D in S1 File). Since plant growth phenomena are never exponential if the entire range of growth is considered, a sigmoid function was used to fit phloem tube number from *n*_*min*_ to *n*_*max*_:

$$n\left(V^{pl}\left(t\right)\right)=n_{min}+\left(n_{max}-n_{min}\right)S_{n}^{+}\left(V^{pl}\left(t\right)\right)$$

where *n*_*min*_(*n*_*max*_) is the minimal (maximal) number of phloem tubes. The parameters of $S_{n}^{+}$ (Fig A in S1 File, panel b) are denoted by *a*_*n*_ and *u*_*n*_.

#### Shoot and root growth

Growth requires sugar for the generation of new building blocks (primarily cell walls). In addition, a certain amount of P_i_ is required per unit of new tissues, reflecting its contribution to membranes and nucleic acids, and its role in diverse metabolic processes. Thus, shoot growth occurs only when the levels of sugar and P_i_ exceed the threshold values $C_{su,gr}^{sh}$ and $C_{ph,gr}^{sh}$, respectively. Maximal growth rate (in cm^3^ per hour) is reached at $g_{max}^{sh}$ when substrate concentrations are high. During plant development, the fraction of shoot volume that can grow is first proportional to the entire shoot volume and ultimately tends to a constant. This reflects the fact that in young plants, all tissues are growing, while at later stages, an increasing proportion of the plant tissues (stem, fully expanded leaves) ceases to grow. This is modeled by the term *M*_*g*,*sh*_ (*V* ^*sh*^ (*t*)). Root growth follows similar laws. These assumptions lead to the equations describing shoot and root growth (Table 2, Eq (1) and Eq (2)):

$${\dot{V}}^{sh}(t)=g_{max}^{sh}\;M_{g,sh}(V^{sh}(t))\;S_{g,sh,su}^{+}(C_{su}^{sh}(t))\;S_{g,sh,ph}^{+}(C_{ph}^{sh}(t))$$ (1)

$${\dot{V}}^{r}(t)=g_{max}^{r}\;M_{g,r}(V^{r}(t))\;S_{g,r,su}^{+}(C_{su}^{r}(t))\;S_{g,r,ph}^{+}(C_{ph}^{r}(t))$$ (2)

where $S_{g,sh,su}^{+},S_{g,r,su}^{+},S_{g,sh,ph}^{+}$ and $S_{g,r,ph}^{+}$ are increasing S-shape functions with parameters $\left(C_{su,gr}^{sh},a_{g,sh,su}\right)$, $\left(C_{su,gr}^{r},a_{g,r,su}\right)$, $\left(C_{ph,gr}^{sh},a_{g,sh,ph}\right)$ and $\left(C_{ph,gr}^{r},a_{g,r,ph}\right)$, respectively. These parameters were estimated by integrating the two above equations and fitting the resulting curves to experimental data (see Fig E in S1 File).

**Table 2:** Terms constituting the mathematical model.

	Description	Equation
1	Leaf growth	${\dot{V}}^{sh}(t)=g_{max}^{sh}\;M_{g,sh}(V^{sh}(t))\;S_{g,sh,su}^{+}(C_{su}^{sh}(t))\;S_{g,sh,ph}^{+}(C_{ph}^{sh}(t))$
2	Root growth	${\dot{V}}^{r}(t)=g_{max}^{r}\;M_{g,r}(V^{r}(t))\;S_{g,r,su}^{+}(C_{su}^{r}(t))\;S_{g,r,ph}^{+}(C_{ph}^{r}(t))$
3	Photosynthesis	$P(t)=P_{\max}\;M_{F,I}(\alpha I(t))\;M_{F,ph}(C_{ph}^{sh}(t))\;S_{photo}^{sh}\;S_{F,st}^{-}(C_{st}^{sh}(t)))\;S_{F,su}^{-}(C_{su}^{sh}(t)))$
4	Sugar Starch partitioning	$\dot{\rho }(t)=-k_{1}\;\rho (t)\;S_{\rho ,1}^{+}(C_{su}^{sh}(t))+k_{2}\;(1-\rho (t))\;D(t)\;S_{\rho ,2}^{-}(C_{su}^{sh}(t))$ $-k_{3}\;\rho (t)\;(1-D(t))\;S_{\rho ,3}^{-}(C_{su}^{sh}(t))$
5	Starch degradation rate	$T_{st\to su}^{sh}(t)=(1-D(t))\;\frac{Q_{st}^{sh}(t)}{L_{night}(t)}\;S_{st}^{-}(C_{su}^{sh}(t))$
6	Leaf respiration	$R^{sh}(t)=g_{R}^{sh}{\dot{V}}^{sh}(t)+(m_{R,1}^{sh}+m_{R,2}^{sh}C_{su}^{sh}(t))V^{sh}(t)+c_{l}^{su}T_{su}^{sh\to r}(t)$
7	Root respiration	$R^{r}(t)=g_{R}^{r}{\dot{V}}^{r}(t)+(m_{R,1}^{r}+m_{R,2}^{r}C_{su}^{r}(t))V^{r}(t)+c_{e}U_{ph}(t)$
8	Solute transport rate from shoot to root compartment	$T_{H_{2}O}^{sh\to r}(t)=\frac{\text{max}\left(C_{su}^{sh}(t)-C_{su}^{r}(t),0\right)}{d_{H_{2}O}^{p}\;R_{tube}(V^{pl}\;(t))/RT}\;n(V^{pl}\;(t))$
9	Sugar transport rate from shoot to root compartment	$T_{su}^{sh\to r}(t)=T_{H_{2}O}^{sh\to r}(t)\;\frac{C_{su}^{sh}(t)}{d_{H_{2}O}^{p}}$
10	P_i_ uptake rate from the soil	$U_{ph}(t)=U_{max}\;M_{U}(C_{ph}^{soil}(t))\;M_{as}(V^{r}(t))$
11	P_i_ transport rate from root to shoot compartment	$T_{ph}^{r\to sh}(t)=\left(E(t)\;S^{sh}(t)+T_{H_{2}O}^{sh\to r}(t)+d_{H_{2}O}^{p}{\dot{V}}^{sh}(t)\right)\;p_{max}^{r}\frac{\text{max}(C_{ph}^{r}(t)-C_{ph,g}^{r},0)}{d_{H_{2}O}^{p}}$
12	P_i_ transport rate from shoot to root compartment	$T_{ph}^{sh\to r}(t)=T_{H_{2}O}^{sh\to r}(t)\;p_{max}^{sh}\frac{\text{max}(C_{ph}^{sh}(t)-C_{ph,g}^{sh},0)}{d_{H_{2}O}^{p}}$

#### Hypotheses and assumptions concerning substrate pools

The carbohydrates from photosynthesis occur either in a soluble form in the shoot and the root (primarily as sucrose and hexoses), or are stored in an insoluble form as starch in the chloroplasts of the shoot [43] (see Fig F in S1 File for further information about the conversion of carbohydrates between the three pools). P_i_ from the soil is taken up by the roots and then redistributed between the shoot and the root, by transpiration stream, and by recycling through the phloem [44]. The following equations reflect the mass balance for the pools of P_i_, soluble carbohydrate, and starch:

Soluble sugar quantity in the shoot:

$${\dot{Q}}_{su}^{sh}\left(t\right)=\rho \left(t\right)P\left(t\right)+T_{st\to su}\left(t\right)-R^{sh}\left(t\right)-\theta_{su}^{sh}{\dot{V}}^{sh}\left(t\right)-T_{su}^{sh\to r}\left(t\right)$$

Soluble sugar quantity in the root:

$${\dot{Q}}_{su}^{r}(t)=T_{su}^{sh\to r}(t)-R^{r}(t)-\theta_{su}^{r}{\dot{V}}^{r}(t)$$

P_i_ quantity in the soil:

$${\dot{Q}}_{ph}^{soil}(t)=-U_{ph}(t)$$

P_i_ quantity in roots:

$${\dot{Q}}_{ph}^{r}(t)=U_{ph}(t)-T_{ph}^{r\to sh}(t)+T_{ph}^{sh\to r}(t)$$

P_i_ quantity in the shoot:

$${\dot{Q}}_{ph}^{sh}(t)=T_{ph}^{r\to sh}(t)-T_{ph}^{sh\to r}(t)$$

where *P*(*t*) is the rate of photosynthesis, $T_{st\to su}^{sh}(t)$ the conversion rate of starch into soluble sugar in the shoot compartment, $\theta_{su}^{sh}$($\theta_{su}^{r}$) the quantity of sugar anabolised to build 1 cm^3^ of shoot (roots), $T_{su}^{sh\to r}(t)$ the sugar transport rate from shoot to root, *R* ^*sh*^(*R* ^*r*^) the shoot (root) respiration rate, *U*_*ph*_ (*t*) the P_i_ uptake rate from the soil, $T_{ph}^{r\to sh}(t)$ the P_i_ transport rate from root to shoot and $T_{ph}^{sh\to r}(t)$ the P_i_ transport rate from shoot to root.

Note that P_i_ is modeled to be necessary within certain concentration boundaries (reflecting its involvement in nucleic acids, membranes etc.), without being incorporated in newly built volume. Thus, P_i_ is not sequestered in a structural pool like fixed carbon. Although real plants fix a certain proportion of P_i_ in nucleic acids and in membranes, these amounts are variable, and P_i_ can be partly remobilized and recycled under P_i_ starvation [45], therefore, all P_i_ is considered to be accessible in our model, in contrast to C which becomes immobilized, primarily as cell walls. A dependence of photosynthesis on P_i_ levels is based on the fact that cytosolic P_i_ is required to balance the export of triose phosphate from the chloroplast by the P_i_:triose phosphate antiporter [46, 47].

#### Hypothesis 6 (Phosphate uptake)

According to [48], P_i_ uptake rate per unit of root absorbing surface corresponds to:

$$U_{max}M_{U}\left(C_{ph}^{soil}\left(t\right)\right)$$

Where *U*_*max*_ is the maximal uptake rate per unit of root active surface and *M*_*U*_ a Monod function with parameter *m*_*U*_. This leads to the following equation for the P_i_ uptake rate *U*_*ph*_ (*t*) (Table 2, Eq (10)), which was used te determine the P_i_-related parameters by fitting to total P_i_ content (Fig G in S1 File):

$$U_{ph}\left(t\right)=U_{max}M_{U}\left(C_{ph}^{soil}\left(t\right)\right)S_{active}^{r}.$$

#### Hypothesis 7 (Photosynthesis)

While photosynthesis is a highly complex biochemical process [49], it is modeled here in a simplified conceptual form. CO_2_ fixation rate $F_{CO_{2}}$ per unit of photosynthetically active leaf surface (in μg CO_2_ cm^−2^ h^−1^) was modeled with the so-called rectangular hyperbola described in [50–53]:

$$P_{max}M_{F,I}\left(\alpha \;I\left(t\right)\right)$$

where *P*_*max*_ is the maximal photosynthesis rate per unit of photosynthetically active leaf surface area (in μg CO_2_ cm^−2^ h^−1^) and *α* denotes the light utilisation efficiency (μg CO_2_ (J PAR)^-1^). *M*_*F*,*I*_ is a Monod function with parameter *m*_*F*,*I*_ = *P*_*max*_. Since several photosynthetic reactions depend on phosphorylation [54], efficient photosynthesis is linked to P_i_ levels in the soluble pool. Furthermore, negative feedback inhibition was introduced at high levels of soluble and stored carbohydrate equivalents, according to experimental evidence [55] (Fig F in S1 File; feedbacks 1 and 6). These feedbacks are represented by the following functions of the soluble and stored carbohydrate concentration and a saturating function of P_i_ concentration:

$$F_{CO_{2}}=P_{max}\;M_{F,I}(\alpha I(t))\;M_{F,ph}(C_{ph}^{sh}(t))S_{F,st}^{-}(C_{st}^{sh}(t)))\;S_{F,su}^{-}(C_{su}^{sh}(t)))$$

where *M*_*F*,*ph*_ is a Monod function with parameter *m*_*F*,*ph*_. $S_{F,st}^{-}$ and $S_{F,su}^{-}$ are decreasing sigmoid functions (Fig A in S1 File, panel c) with parameters $\left(s_{F,st},sd_{F,st},C_{st,max}^{sh}\right)$ and $\left(s_{F,su},sd_{F,su},C_{su,max}^{sh}\right)$, respectively.

#### Hypothesis 8 (Rate of photosynthesis)

The rate of photosynthesis *P*(*t*) (in μg sugar per hour) is equal to the product of the CO_2_ fixation rate $F_{CO_{2}}$ per unit of photosynthetically active leaf surface area and the photoactive leaf surface $S_{photo}^{sh}$ (see hypotheses 2 and 7 above):

$$P\left(t\right)=c_{1}\cdot F_{CO_{2}}\cdot S_{photo}^{sh}$$

where *c*_1_ is a unit conversion constant.

#### Hypothesis 9 (Fraction of carbohydrate stored as starch)

As shown in the scheme Fig F in S1 File, carbohydrate units produced by photosynthesis in the chloroplast can be either stored as starch or transported directly to the cytosol (soluble pool) [43, 56]. Carbohydrate stored during the day is used for maintenance metabolism (respiration) and growth at night. We denote by *ρ* the fraction of photosynthate directly transferred to the soluble sugar pool. As in *Arabidopsis thaliana* [57] grown under standard conditions (L:D, 12h:12h), approximatively half of the reduced carbon is stored immediately after photosynthesis in the model [47]. In agreement with the regulation of starch synthesis by ADP-glucose-pyrophosphorylase (reviewed in [56, 58]), the fraction *ρ* depends primarily on soluble carbohydrate concentration.

#### Hypothesis 10 (Regulation of the fraction of carbohydrate stored as starch)

Soluble carbohydrate concentration is kept within narrow limits during day and night [43, 56], i.e. near a physiological target concentration $C_{su,t}^{sh}$. If carbohydrate levels in the soluble pool exceed this value during the day, the partitioning coefficient *ρ* decreases (Fig F in S1 File, feedback 2), whereas it increases in case of carbohydrate starvation (that is when the carbohydrate levels in the cytosol are below the critical value $C_{su,t}^{sh}$ during the day (Fig F in S1 File, feedback 4). However, carbohydrate starvation at night will decrease *ρ* [47], so that carbohydrate storage will be favored at the beginning of the following day (Fig F in S1 File, feedback 5). This reflects an adaptive mechanism of the plant to increase the storage pool during the subsequent light period to avoid repeated sugar starvation during the next night period [59]. The following equation for the partitioning between starch and soluble sugar reflects this mechanism:

$$\dot{\rho }(t)=-k_{1}\;\rho (t)\;S_{\rho ,1}^{+}(C_{su}^{sh}(t))+k_{2}\;(1-\rho (t))\;D(t)S_{\rho ,2}^{-}(C_{su}^{sh}(t))$$

$$-k_{3}\;\rho (t)\;(1-D(t))\;S_{\rho ,3}^{-}(C_{su}^{sh}(t)),$$

where the first term is the adaptation to high soluble carbohydrate concentration during the day, the second one the increase of *ρ* under carbohydrate starvation during the day and the last one the increase of *ρ* when sugar starvation occurs during the night. The values of parameters *k*_1_, *k*_2_ and *k*_3_ lay between 0 and 1. $S_{\rho ,1}^{+}$, $S_{\rho ,2}^{-}$ and $S_{\rho ,3}^{-}$ are sigmoid functions with parameters $\left(a_{\rho ,1},C_{su,t}^{sh}\right)$, $\left(s_{\rho ,2},sd_{\rho ,2},C_{su,t}^{sh}\right)$ and $\left(s_{\rho ,3},sd_{\rho ,3},C_{su,t}^{sh}\right)$, respectively.

#### Hypothesis 11 (Starch degradation)

Stored carbohydrate in the starch pool is degraded during the night phase [43] and transferred with the rate $T_{st\to su}^{sh}(t)$ to the soluble pool. In agreement with experimental evidence, the rate of starch degradation is proportional to the storage pool size, and proceeds linearly during the night phase [12]. This implies a mechanism in the plant to predict the length of the night. It is provided by an internal clock [60–62], represented here by an internal oscillator with period *δ*_*IO*_ (for Petunia: *δ*_*IO*_ = 25 hours) supposed to be synchronized each day with the day-and-night cycle at the beginning of the day. In summary, the rate of starch degradation $T_{st\to su}^{sh}(t)$ is modeled by

$$T_{st\to su}^{sh}(t)=(1-D(t))\;\frac{Q_{st}^{sh}(t)}{L_{night}(t)}\;S_{st}^{-}(C_{su}^{sh}(t))$$

where *L*_*night*_ (*t*) is the expected time until the end of the night and is equal to *δ*_*IO*_ minus the actual time since the beginning of the day. $S_{st}^{-}$ is a decreasing sigmoid function with parameters $\left(s_{st},sd_{st},C_{su,t}^{sh}\right)$. The latter is a negative feedback to attenuate starch degradation at high soluble carbohydrate concentrations (Table 2, Eq (5); Fig F in S1 File, feedback 3).

#### Hypothesis 12 (Sugar transport from the shoot to the root)

Translocation of carbohydrate from the shoot (source) to the root (sink) proceeds through the vascular strands within the phloem (see hypotheses 4 and 5 above). The phloem in the model consists of impermeable tubes which are semi-permeable at both ends for carbohydrate loading and unloading. The mechanism, suggested by Münch [63], is known as mass flow process and generally agreed to mediate long-distance phloem transport of herbaceous plants [39]. Solute flux through individual tubes is modeled by the osmotically driven Poiseuille flow [22, 64]. It depends on the difference between soluble sugar concentrations in the source and sink region respectively-$C_{su}^{source}\left(t\right)\;(C_{su}^{sink}\left(t\right)$)- and the tube resistance *R*_*tube*_. Solute flux from the shoot to the root $T_{H_{2}O}^{sh\to r}\left(t\right)$ (Table 2, Eq (8)) is thus obtained by multiplying solute flux through one phloem tube with the number of tubes *n*(*V* ^*pl*^ (*t*)):

$$T_{H_{2}O}^{sh\to r}(t)=\frac{\text{max}\left(C_{su}^{source}(t)-C_{su}^{sink}(t),0\right)}{d_{H_{2}O}^{p}\;R_{tube}(V^{pl}(t))/RT}\;n(V^{pl}(t))$$

where $d_{H_{2}O}^{p}$ is the quantity of solute per cm^3^ of plant, *T* the temperature and *R* the gaz constant. Phloem tube resistance *R*_*tube*_ is given by:

$$R_{tube}\;(V^{pl}\;(t))=c_{2}\frac{8L(V^{pl}\;(t))\eta }{\pi r^{4}}$$

where *η* is the viscosity, *r* the average phloem tube radius (in cm) and *c*_*2*_ a unit conversion parameter. The parameters: $d_{H_{2}O}^{p}=0.9250\;cm^{3}\;H_{2}0\;cm^{-3}$ plant, *T* = 295 K, *R* = 1.078 ⋅10^15^ g cm^2^ h^-2^ mol^-1^ K^-1^, *η* = 108 *g cm* ^−1^ *h* ^−1^ [65] and *c*_2_ = 5.56⋅10^−9^ are known. The last one, *r*, is of the order of 10^−3^ cm [64].

#### Hypothesis 13 (Sugar concentrations in the phloem)

Mass flow through the phloem is driven by phloem loading in the photosynthetic source leaves [39]. Therefore, and in agreement with previous modeling studies [26, 36, 66], we assume that local carbohydrate concentration in the source region of the phloem ($C_{su}^{source}$) corresponds to carbohydrate concentration in the leaves. The same assumption holds for the sink concentration ($C_{su}^{sink}$). To account for the active components in phloem transport [39], costs for carbohydrate transfer were introduced.

#### Hypothesis 14 (Rate of sugar transport between shoot and root)

The transfer rate of carbohydrates from the shoot to the root depends both on their production in the source leaves (by photosynthesis), and on their use in the sink tissues [67]. This fact is reflected in the model by the dependence of solute transport on sugar concentration in both, the root and the shoot, and on the resulting difference (concentration gradient) between the two (see hypothesis 12). The carbohydrate quantity transferred from the shoot to the root per unit of time $T_{su}^{sh\to r}\left(t\right)$ (Table 2, Eq (9)) corresponds to solute flux $T_{H_{2}O}^{sh\to r}\left(t\right)$ (in cm^3^ per hour) multiplied with the carbohydrate concentration in the tube at the source region (identical to $C_{su}^{sh}\left(t\right)$):

$$T_{su}^{sh\to r}(t)=T_{H_{2}O}^{sh\to r}(t)\;\frac{C_{su}^{sh}(t)}{d_{H_{2}O}^{p}}$$

#### Hypothesis 15 (Water flux from the root to the shoot)

Water and mineral nutrients are transported by the transpiration stream in the xylem [68]. Water flux from the root to the shoot through the xylem $T_{H_{2}O}^{r\to sh}\left(t\right)$ is composed of the volume of water transpired by the leaves, the replacement for the volume of solute transported downwards in the phloem, ($T_{H_{2}O}^{sh\to r}\left(t\right)$), and the volume of water incorporated in newly formed tissues. Since the water content per cm^3^ of plant $d_{H_{2}O}^{p}$ is assumed to be constant, we have the following balance equation for water in the shoot:

$$d_{H_{2}O}^{p}{\dot{V}}^{sh}(t)=T_{H_{2}0}^{r\to sh}(t)-E(t)\;S^{sh}(t)-T_{H_{2}O}^{sh\to r}(t)$$

and thus:

$$T_{H_{2}0}^{r\to sh}(t)=E(t)\;S^{sh}(t)+T_{H_{2}O}^{sh\to r}(t)+d_{H_{2}O}^{p}{\dot{V}}^{sh}(t)$$

where $S^{sh}(t)=\frac{2V^{sh}(t)}{th(J)}$ is the leaf surface area and *E*(*t*) the transpiration rate per unit of leaf surface area. The latter is denoted *E*_*d*_ during the day, *E*_*n*_ during the night and

$$E\left(t\right)=E_{n}+\left(E_{d}-E_{n}\right)D\left(t\right)$$

provides a continuous extension of *E*(*t*) during the transition phases.

#### Hypothesis 16 (Phosphate transport from the root to the shoot)

P_i_ is transported from the root to the shoot via the transpiration stream [69] (see also hypothesis 15). The P_i_ quantity transported from the root to the shoot in the xylem per unit of time corresponds to the solute flux through the xylem $T_{H_{2}O}^{r\to sh}\left(t\right)$ multiplied with P_i_ concentration in the xylem sap. The latter is proportional (with the coefficient $p_{max}^{r}$) to P_i_ concentration in the root minus the critical P_i_ concentration $C_{ph,g}^{r}$ that is held back in the root for its own use, and therefore cannot be translocated (see Table 2, Eq (11)):

$$T_{ph}^{r\to sh}(t)=T_{H_{2}0}^{xylem}(t)\cdot p_{max}^{r}\frac{\text{max}(C_{ph}^{r}(t)-C_{ph,g}^{r},0)}{d_{H_{2}O}^{p}}$$

#### Hypothesis 17 (Phosphate transport from the shoot to the root)

A certain proportion of P_i_ is cycled back from the shoot to the root through the phloem [70, 71]. This P_i_ flux corresponds to the volume of solution transported in the phloem $T_{H_{2}O}^{sh\to r}\left(t\right)$ (see hypothesis 12) multiplied with the P_i_ concentration in the phloem. The latter is assumed to be proportional (with coefficient $p_{max}^{sh}$) to the P_i_ concentration in the shoot minus the critical P_i_ concentration $C_{ph,g}^{sh}$ that is the minimal concentration of phosphate required per unit of plant volume (see Table 2, Eq (12)).

$$T_{ph}^{sh\to r}(t)=T_{H_{2}O}^{sh\to r}(t)\cdot p_{max}^{sh}\frac{\text{max}(C_{ph}^{sh}(t)-C_{ph,g}^{sh},0)}{d_{H_{2}O}^{p}}$$

#### Hypothesis 18 (Costs of respiration)

As in previous models [72–75], respiration reflects the sum of the energy-consuming processes involved in the growth of new tissue and in the maintenance of the existing one. In addition, active transport contributes to respiration [76]. The respiration rate in the shoot *R* ^*sh*^ (*t*) (Table 2, Eq (6)) and the root *R* ^*r*^ (*t*) (Table 2, Eq (7)) corresponds to the sum of maintenance respiration ($R_{m}^{sh}$(t)and $R_{m}^{r}\left(t\right)$), growth respiration ($R_{g}^{sh}\left(t\right)$and $R_{g}^{r}\left(t\right)$) and transport costs ($R_{t}^{sh}\left(t\right)$and $R_{t}^{r}\left(t\right)$):

$$R^{sh}(t)=R_{m}^{sh}(t)+R_{g}^{sh}(t)+R_{t}^{sh}(t)\;\text{and}\;R^{r}(t)=R_{m}^{r}(t)+R_{g}^{r}(t)+R_{t}^{r}(t)$$

As suggested by [77, 78], maintenance respiration per unit of volume is assumed to be a linear function of soluble carbohydrate concentration:

$$R_{m}^{sh}(t)=(m_{R,1}^{sh}+m_{R,2}^{sh}C_{su}^{sh})V^{sh}(t)\;\text{and}\;R_{m}^{r}(t)=(m_{R,1}^{r}+m_{R,2}^{r}C_{su}^{r})V^{r}(t)$$

Growth respiration is proportional to growth rate:

$$R_{g}^{sh}(t)=g_{R}^{sh}{\dot{V}}^{sh}(t)\;\text{and}\;R_{g}^{r}(t)=g_{R}^{r}{\dot{V}}^{r}(t)$$

Relevant transport processes include, among others, P_i_ uptake into root epidermis and phloem loading with photosynthates in the shoot. Thus:

$$R_{t}^{sh}(t)=c_{l}^{su}T_{su}^{sh\to r}(t)\;\text{and}\;R_{t}^{r}(t)=c_{e}U_{ph}(t)$$

### Model implementation and parameter estimation

The model was implemented in C and compiled in Matlab. For integration, the Runge-Kutta method was used with a time step of 0.003 h (10.8 s), with *t* = 0 corresponding to the time of sowing. The initial states of the nine state variables for the simulations in each experiment are listed in Table A in S1 File. The model contains a total of 55 parameters that were estimated in two steps as follows: In a first step, 29 parameters were determined experimentally or fitted as part of the submodels (Figs B-E and G in S1 File). Among these, 21 parameters were fixed (S1 Table). The other 8 parameters were fitted again in a second round together with the remaining 26 parameters to result in an additional set of 34 parameters (S2 Table). The initial values for 10 of the set of 26 parameters were derived from the literature [51, 64, 65, 76, 78–81], whereas for the remaining 16 parameters the initial value was estimated tentatively. Finally, the 34 unfixed parameters (S2 Table) were subjected to a global fit against a set of experimental data (experiment 1; experiment 2, treatments A and B) using the Nelder-Mead method, allowing them to vary within a predefined biological interval (S2 Table) of approximately +/- 10% around the initial value. The other intervals can be found in S2 Table. Note that most of the parameters determined by global fitting (S2 Table) are related to growth, starch dynamics, photosynthesis, respiration and P_i_ transport, whereas all the parameters associated with P_i_ uptake and most of those in sugar transport were fixed (S1 Table).

### Implementation of the model of Thornley (1998b)

Thornley’s model consists of 6 state variables (Table A in S2 File), and 17 parameters (S3 Table). For further detail (mathematical equations and notations), the reader is referred to [24]. Thornley’s model was implemented in C and compiled in Matlab. Parameters were estimated in two steps. A first set of 9 parameters was determined experimentally or by fitting submodels to experimental data as it was done for our model. Among the remaining parameters, 5 were derived from the literature [24, 51] and 3 were estimated tentatively.

In a second step, a global fitting of all the parameters was performed by varying them in a neigborhood of the estimated values (see S3 Table) and fitting the resulting simulations to the same subset of experimental data as in the case of our model (experiment 1; experiment 2, treatments A and B) using the Nelder-Mead method.

### Comparison of the two models with Pareto fronts

At the structural level, Thornley's and our model are similar, i.e. they both consist of two compartments (the shoot and the root), and involve the balanced exchange of the substrates sugar and P_i_ between these compartments. Photosynthesis and P_i_ uptake are modeled similarly in both models. However, the two models differ in the following aspects. (i) In contrast to Thornley, we have introduced general costs for growth as well as for uptake and transport processes. (ii) Sugar and P_i_ transport are mediated by diffusion in Thornley’s model, while ours invokes mass flow; (iii) Thornley's model involves continuous light, whereas ours has a day-and-night cycle; (iv) In contrast to Thornley's, our model has a transitory carbohydrate storage compartment (starch) and a circadian clock as adaptations to the day-and-night cycle; (v) In our model, shoot and root growth are 0 if sugar or P_i_ contents fall below a critical threshold concentration, whereas in Thornley's model, plant growth is proportional to sugar and P_i_ levels; and finally (vi) the photosynthetically active surface area depends on the light intensity in our model in contrast to Thornley's model.

**Fig 3:**
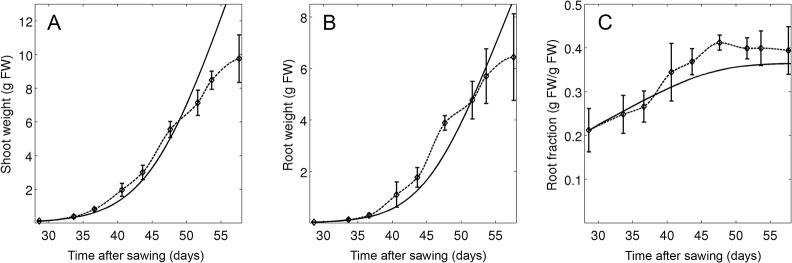
Parameter fitting at optimal growth conditions. Plants were grown at high light levels (595 μmol m^-2^ s^-1^) and a saturating P_i_ concentration in the soil (300 μM). Simulations (continuous line) and experimental data (Experiment 1; dashed line) are shown for shoot growth (A), root growth (B), and the relative root fraction (C). Error bars represent the standard deviations (N = 6).

**Fig 4:**
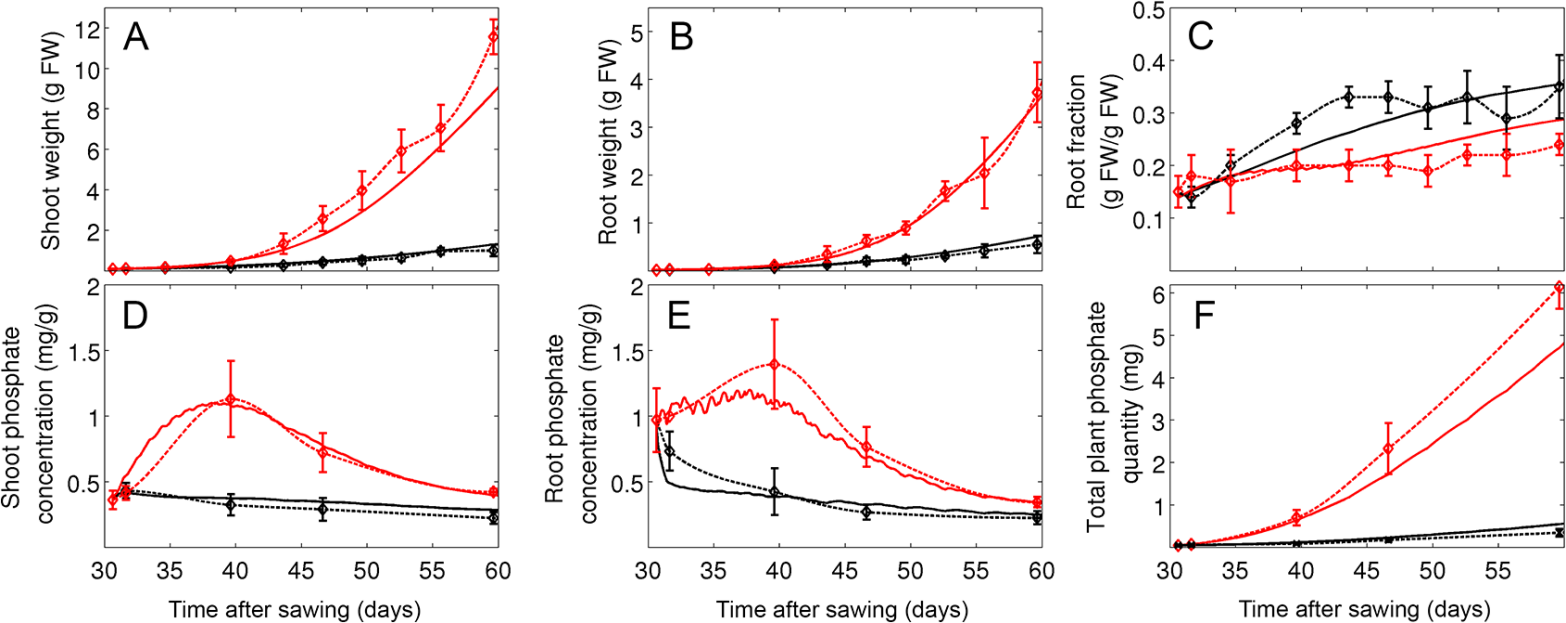
Parameter fitting under two different phosphate levels. Plants were grown at an intermediate light level (316 μmol m^-2^ s^-1^) and at two P_i_ regimes representing limiting conditions (10 μM, black curves) and intermediate conditions (100 μM, red curves). Simulations (continuous lines) and experimental data (Experiment 2, treatments A and B; dashed lines) are shown for shoot weight (A) and root weight (B), root fraction (C), P_i_ levels in shoot (D) and in root (E) and total P_i_ in plants (F). Error bars represent the standard deviations (N = 5).

**Fig 5:**
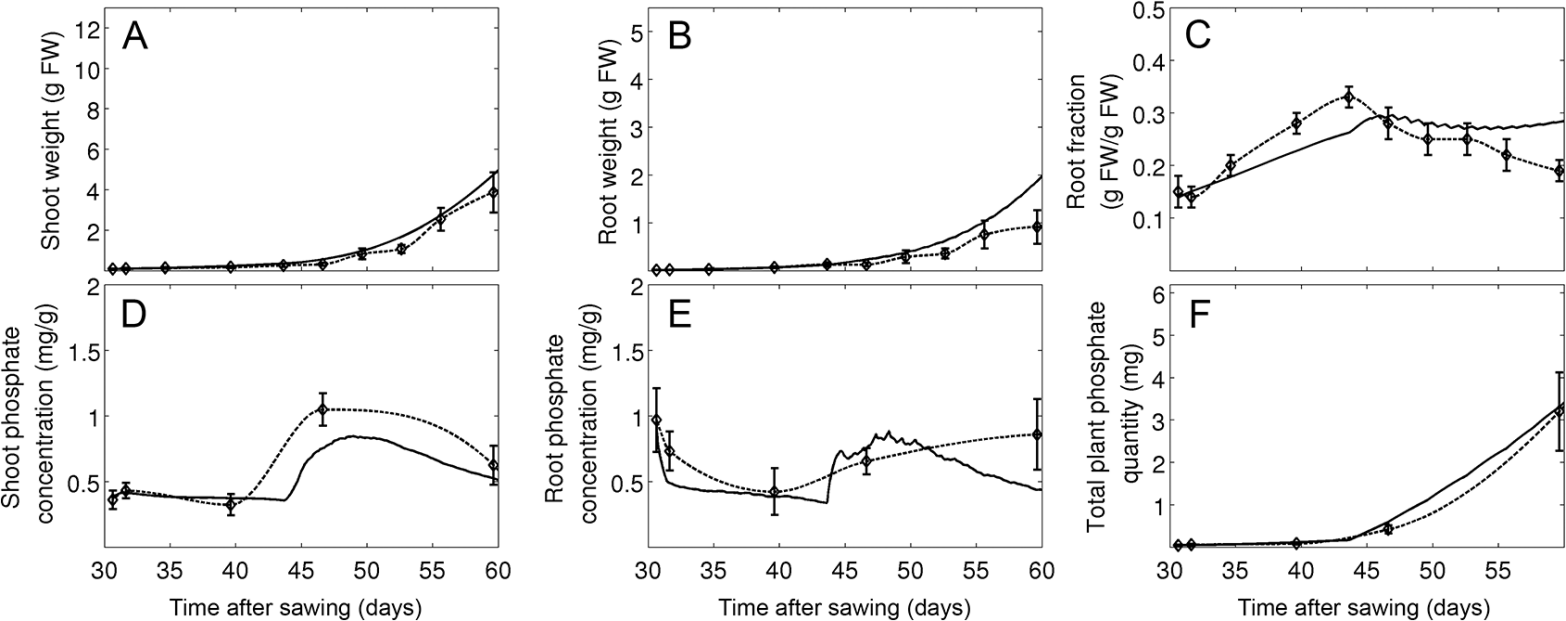
Model validation and evaluation of adaptive potential of shoot and root growth towards increasing P_i_ supply. Plants were first grown at low P_i_ levels (10 μM), followed by a switch to 100 μM after two weeks. Simulations (continuous lines) and experimental data (Experiment 2, treatment C; dashed lines) are shown for shoot weight (A), root weight (B), root fraction (C, P_i_ levels in the shoot (D) and in the root (E) and total P_i_ per plant (F). Error bars represent the standard deviations (N = 5).

**Fig 6:**
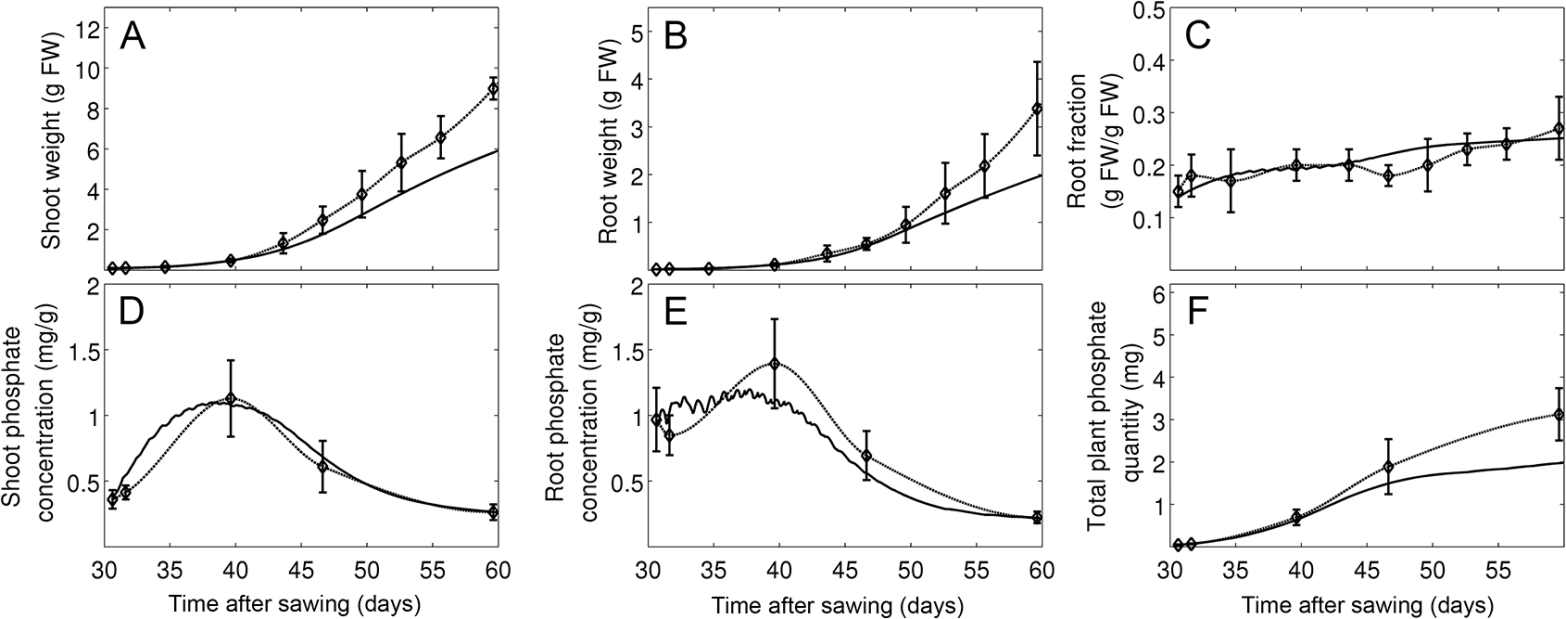
Model validation and evaluation of adaptive potential of shoot and root growth towards decreasing P_i_ supply. Shoot and root growth was analyzed after a switch from high P_i_ levels (100 μM) to 10 μM after two weeks (reversed switch compared to Fig 3). Simulations (continuous lines) and experimental data (experiment 2, treatment D; dashed lines) are shown for shoot weight (A) and root weight (B), root fraction (C), P_i_ levels in the shoot (D) and the root (E) and total P_i_ per plant (F). Error bars represent the standard deviations (N = 5).

**Fig 7:**
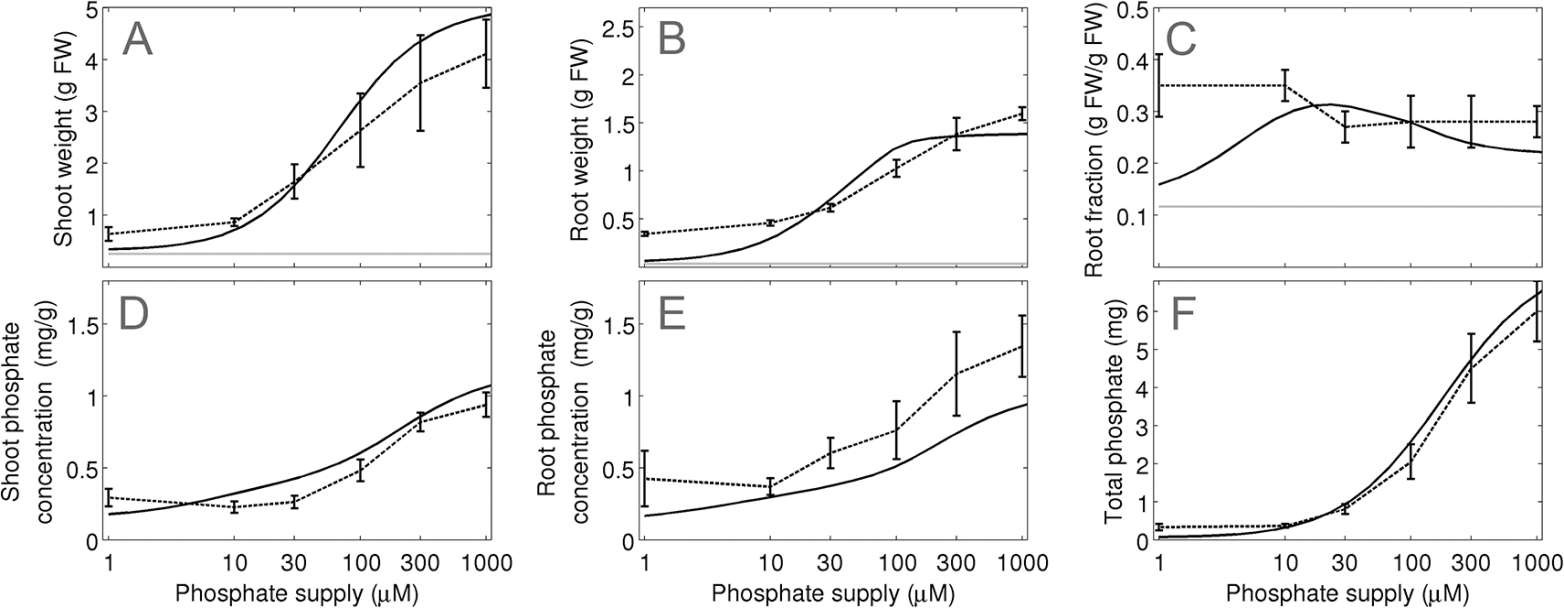
Model validation and evaluation of the adaptive potential of plants to a range of different P_i_ concentrations. Simulations (continuous line) and experimental data (experiment 3; dashed line) are shown for (A) shoot and (B) root growth, (C) root fraction, (D) shoot P_i_ concentration and (E) root P_i_ concentration and (F) total P_i_ per plant for plants grown at 6 different P_i_ concentrations (1, 10, 30, 100, 300, 1000 μM). The grey line in (a)-(c) represents the value at the beginning of the experiment. Error bars represent the standard deviations (N = 10).

For an initial comparison of the two models, Thornley's model was used to simulate the experimental results as with ours (Figs A-H in S2 File; compare with Figs 3–7). In order to compare the performance of the two models in a quantitative way, Pareto fronts were calculated for three pairs of variables, namely shoot and root volume (*V* ^*sh*^(*t*),*V* ^*r*^(*t*)), shoot and root P_i_ concentration $\left(C_{ph}^{sh}(t),C_{ph}^{r}(t)\right)$, and shoot and root sugar concentration $\left(C_{su}^{sh}(t),C_{su}^{r}(t)\right)$. For each parameter pair, the sum of the relative quadratic errors (RE) between simulated and observed values was determined, denoted by *RE*_*V*_, *RE*_*ph*_, and *RE*_*su*_, respectively. For the calculation of *RE*_*su*_ in our model, the mean soluble sugar concentration over 24 hours was used, since the sugar oscillations brought about by the day-and-night cycle in our model would make the comparison with Thornley’s model difficult. Calculation of the Pareto front involves the minimization of the components (*RE*_*V*_, *RE*_*ph*_, *RE*_*su*_) by changing the parameter sets of the two models after separate fitting. One way to compare the two models is to calculate the weighed sum of *RE*_*V*_, *RE*_*ph*_, and *RE*_*su*_. This procedure (Nelder-Mead approach) results in a single number that may be sensitive to the initial condition and therefore is of limited use. To circumvent this problem, another optimization method, a so-called genetic algorithm, was chosen and implemented in matlab (function gaoptimset.m of the global optimization toolbox). For a detailed description of the procedure, the reader is referred to [82]. This approach minimizes independently the three relative errors *RE*_*V*_, *RE*_*ph*_, and *RE*_*su*_. Instead of yielding a single optimal set, as it is the case with the Nelder-Mead approach, the genetic algorithm provides several optimal sets. The smaller the values for all the three criteria (*RE*_*V*_, *RE*_*ph*_, *RE*_*su*_) are, the better a given parameter set is. Keeping only the best points leads to a collection of different optimized points (*RE*_*V*_, *RE*_*ph*_, *RE*_*su*_) called the Pareto front.

## Results

### Adaptive regulation of root:shoot ratio in *Petunia hybrida*

To explore the adaptive potential of *P*. *hybrida* in resource partitioning between the shoot and the root, we exposed young plants to different light intensities and phosphate (P_i_) levels, which alter the respective service of the shoot and the root. First, plants were grown at three different light intensities *J* (450 μmol m^−2^ s^−1^, 191 μmol m^−2^ s^−1^ and 93 μmol m^−2^ s^−1^) and supplemented with high P_i_ levels (300 μM). As expected, lower light intensities resulted in a lower root fraction (RF), defined as the root fresh weight divided by the fresh weight of the entire plant (Fig 1A, S4 Table). Hence, plants compensated reduced photosynthetic performance due to insufficient light by stimulating shoot growth relative to the root. In addition, reduced light caused the leaves to become thinner, resulting in a specific expansion of the photosynthetic area at the expense of leaf thickness (Fig B in S1 File).

Secondly, the adaptive behavior of *P*. *hybrida* towards different levels of P_i_ was assessed. Plants were grown under a light intensity *J* of 316 μmol m^−2^ s^−1^ at low (10 μM) or high (100 μM) levels of KH_2_PO_4_, resulting in an adaptive response where low P_i_ supply preferentially stimulated root growth, relative to the shoot (Fig 1B). Interestingly, if the P_i_ regimes were swapped during the experiment (after 41 days), the plants dynamically readjusted the root fraction to the new P_i_ levels (Fig 1B).

### Modeling of resource partitioning and plant growth

In order to address the relative growth of the shoot and the root and their interactions in a systematic and integrated way, we developed a mechanistic mathematical model that includes submodels for photosynthesis, nutrient uptake and transport, and which is embedded in a realistic environment. The details of model structure and functioning, and the determination of its parameters, are described in Materials and Methods, and in the Supporting Information. Below, we provide a brief description of the model and its validation. The model comprises two main compartments, the shoot and the root (Fig 2). The shoot produces carbohydrates by photosynthesis, whereas the root acquires water and nutrients (represented here by P_i_) from the substrate. Soluble sugar can be either invested in shoot growth (structure), or transferred to the heterotrophic root through the phloem. The root, on the other hand, transfers P_i_ to the shoot through the xylem. New building blocks in the root and the shoot are produced if sugar and P_i_ reach a defined permissive threshold. To enable the virtual plant to survive in a variable environment with a day-and-night cycle, a transitory carbohydrate storage pool (starch) and an internal oscillator (circadian clock) had to be introduced. The model contains several submodels for photosynthesis, nutrient uptake and transport, and consists of a total of nine state variables that represent the volumes of the shoot and the root, and their P_i_ and sugar content (Table 1). For further detail, the reader is referred to the Materials and Methods section, and to the Supporting Information.

### Parameter fitting and model validation

The model contains a total of 55 parameters that were defined by experimental determination and fitting (see Materials & Methods and S1 and S2 Tables) against a dedicated set of experimental data (experiment 1; experiment 2, treatments A and B). The fitting resulted in a good match between experimental data and simulations concerning the development of shoot and root weight and root fraction under favorable growth conditions, although growth was in general overestimated at late time points (experiment 1; Fig 3). Similarly, a time course experiment carried out at low (10 μM) and high (100 μM) P_i_ concentrations (experiment 2, treatments A and B), resulted in a good match of experimental data and simulations for weight and P_i_ content of the shoot (Fig 4A and 4D) and the root (Fig 4B and 4E), as well as for root fraction (Fig 4C) and total plant P_i_ content (Fig 4F), although the latter deviated at the latest time point of the high P_i_ treatment.

The model was then validated with an independent set of experimental data, that had not been used for parameter fitting. First, we explored the dynamics of the adaptive potential of petunia under changing P_i_ conditions (experiment 2, treatments C and D). In this experiment, plants were first grown at low (10 μM) or high (100 μM) P_i_ concentrations for two weeks, followed by a respective swap of the P_i_ supply regime (Figs 5 and 6). In the case of the decrease from 100 μM to 10 μM, the change in P_i_ concentration in the model was performed gradually over a period of five days to reflect the delayed depletion of P_i_ from transiently adsorbed pools on sand particles.

The relative growth of the root and the shoot (i.e. root fraction) is sensitive to P_i_ supply (Figs 1B and 4), and can therefore be expected to be dynamically regulated. Indeed, plants grown first at low P_i_ levels showed an initial increase in root fraction, followed by a decrease after the switch to high P_i_ levels, and a similar, though slightly less pronounced trend, was observed in our simulations (Fig 5C). The experimental data as well as the simulations for P_i_ levels in the shoot (Fig 5D) and the root (Fig 5E), as well as total P_i_ content of the plant (Fig 5F) reflected the increase in P_i_ supply at day 41. The reverse swap experiment (first high, then low P_i_ levels) caused only a small increase of root fraction after the change (Fig 6C), presumably because accumulation of P_i_ during the first phase at high P_i_ levels (Fig 6D and 6E) allowed the plants to acquire and store enough P_i_ for sustained development during the subsequent phase at low P_i_ levels, and this behavior was also reflected in our simulations.

Sugar levels in the model oscillated with a diurnal period in a narrow band between 0.5 and 1.5 mg/g (Fig H in S1 File), in a similar range as experimental values. In general, simulated sugar levels were slightly higher at low P_i_ (Fig H in S1 File, panel a) compared to high P_i_ levels (Fig H in S1 File, panel b), a trend that was also observed in the swap experiments from low to high P_i_ (Fig H in S1 File, panel c), and, to a lesser extent, from high to low P_i_ levels (Fig H in S1 File, panel d). Deviations between predicted and measured sugar levels were found only at high P_i_ levels, where measured sugar levels raised (Fig H in S1 File, panel b), and after the switch from high to low P_i_ levels, when sugar levels decreased below the predicted values (Fig H in S1 File, panel d).

The second data subset (experiment 3) was assigned to test the adaptive potential of relative shoot and root growth towards a wider range of P_i_ levels. Plants were grown at P_i_ concentrations between 1 μM and 1 mM and harvested at a single time point (Fig 7). Consistent with the previous experiments, the model predicted an elevated root fraction at low P_i_ supply (Fig 7C) but only in the range between 10 μM and 30 μM. At the extremely low P_i_ concentration of 1 μM, the virtual plant hardly grew (Fig 7A and 7B) and only a moderate increase of RF was observed relative to the initial state (Fig 7C, grey line). However, simulations and measurements between the P_i_ levels in the shoot and the root as well as P_i_ content of the entire plant were in good agreement. This difference in growth and shoot ratio between simulations and experimental data at very low P_i_ concentrations may be due to compensatory P_i_ starvation mechanisms of plants that are not represented in the model.

### Assessing the roles of the submodels and of exogenous cues in the global behaviour of the model

With the encouraging results from parameter fitting and model validation, we set out to test the roles of individual components of the model. This was achieved by eliminating or varying the submodels and analysing the resulting effects, which reveal their role in the emergent behaviour of the model as a whole. *In silico* "knock-out" analysis of carbohydrate storage and of the endogenous clock revealed an absolute necessity for both components. The virtual plant died during the first night (without storage), or soon after (without the clock), due to depletion of the sugar pool during the night (data not shown).

In order to test the role of the clock in the adaptation to photoperiod, the setting of the model was changed from 12 h day and 12 h night (12:12) to 10:10, 14:14, and 16:16 photoperiod, respectively to test for sensitivity of the system to the total length of photoperiod (Figs I-K in S1 File). Extended photoperiod inhibited shoot growth (Fig I in S1 File, panel a), whereas the shortened photoperiod inhibited root growth (Fig I in S1 File, panel b), resulting in pronounced changes of root fraction (Fig I in S1 File, panel c). More importantly, shortened photoperiod caused starch to be only partially used during the night (Fig J in S1 File, panel a, compare with Fig J in S1 File, panel b), whereas extended photoperiods led to depletion of the starch reserves (Fig J in S1 File, panels c,d). Interestingly, soluble sugar concentrations remained buffered under 10:10 photoperiod within similar limits as under 12:12 conditions (Fig K in S1 File, panels a,b). However, sugar levels oscillated with increasing amplitude at elevated photoperiod, in particular under 16:16 conditions where sugar levels became completely depleted during the nights, resulting in the death of the plant (Fig K in S1 File, panels c,d).

**Fig 8:**
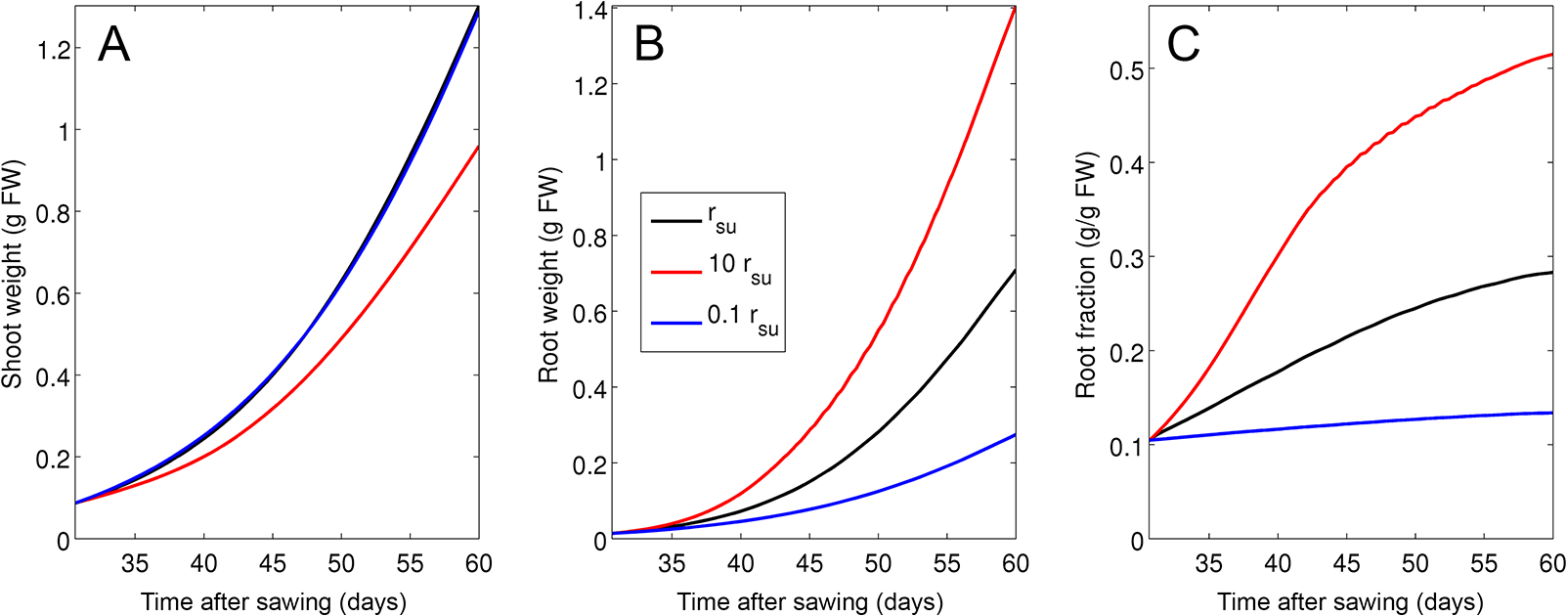
Simulation of growth dynamics as a function of phloem resistance. Shoot (A) and root (B) weight was simulated in conceptual mutants with decreased or increased phloem resistance (*r*_*su*_) by a factor 0.1 and 10, respectively. Root fraction (C) was decreased by transport deficiency (blue line; factor 0.1), and increased by stimulated transport (red line; factor 10) relative to the control (black line).

Next, we simulated plants in which phloem resistance was increased or decreased by 10-fold, respectively (Fig 8). Such changes had little effect on shoot growth (Fig 8A), however, root growth responded very strongly (Fig 8B), leading to a much lower root fraction when phloem resistance was increased, whereas a decrease in phloem resistance increased the root fraction (Fig 8C).

**Fig 9:**
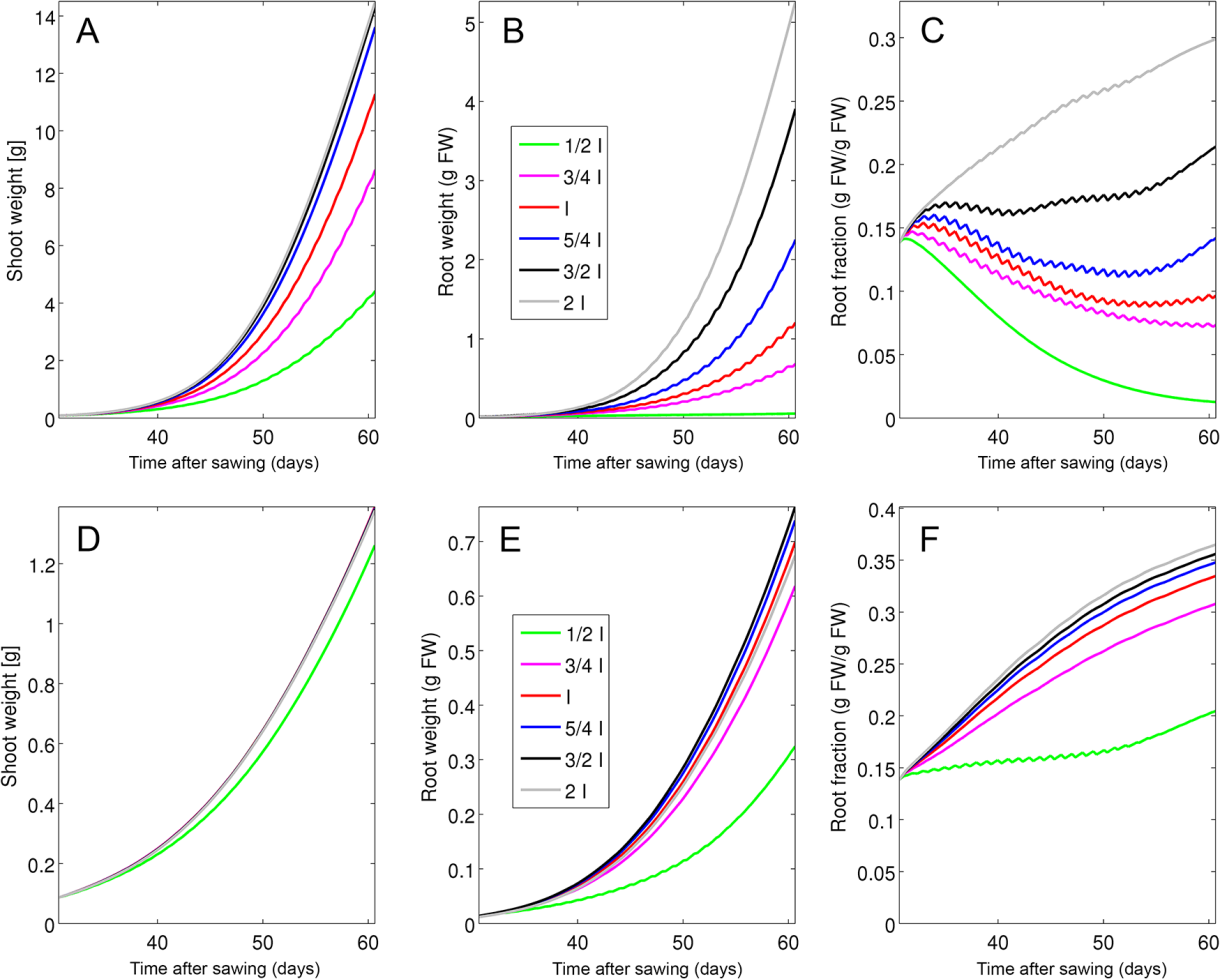
Simulation of the competing effects of limited light irradiance and P_i_ starvation on plant growth and root fraction. Growth of the shoot (A,D) and the root (B,E), as well as the resulting root fraction (C,F) are given for six light levels between 100 and 400 μmol m^-2^ s^-1^ (I = 200 μmol m^-2^ s^-1^) at high P_i_ levels of 300 μM (A-C) and P_i_ starvation conditions at 10 μM (D-F).

Next we tested the relative sensitivity of the model to combined changes in the exogenous cues light and phosphate under yet unexplored conditions. We first simulated growth at various light conditions from 100 μmol m^−2^ s^−1^ to 400 μmol m^−2^ s^−1^, and these simulations were carried out at two different P_i_ levels of 10 μM and 300 μM, corresponding to P_i_ starvation and to saturating P_i_ levels, respectively. At high P_i_ supply, low light levels (100 and 200 μmol m^−2^ s^−1^) caused shoot growth to become decreased (Fig 9A), however, the effect on root growth was much more dramatic (Fig 9B), leading to strong reductions of root fraction at all light levels below the maximum of 400 μmol m^−2^ s^−1^ (Fig 9C). If plants were in addition exposed to P_i_ starvation, the inhibiting effect of low light on root growth was much less pronounced (Fig 9E), and the resulting decrease in root fraction was weaker (Fig 9F). These results show that the model exhibits realistic global behaviour under combined environmental changes of light and nutrients.

### Comparison with Thornley’s model

The focus of our model is on the dynamics of resource allocation. In order to evaluate the performance of this feature relative to previous models, we compared it with the standard model of Thornley [24], that exhibits many similarities to ours, but differs in some submodels (see 2.2.4), and which does not involve a day-and-night cycle (see Materials and Methods and Discussion for further detail). With a parameter set fitted to our experimental data set (S3 Table), Thornley’s model provided good results with the environmental conditions of experiment 1 and experiment 2 (treatments A and B), particularly when plants under favorable conditions were considered (Fig E in S2 File, compare with Fig 3). Unexpectedly, however, when the growth conditions with high and low P_i_ supply were compared, root fraction increased with high instead of low P_i_ levels (Fig F in S2 File, panel c; compare with Fig 4C), whereas the P_i_ levels in the shoot and the root, as well as total P_i_ content showed a good match (Fig F in S2 File, panels d-f). Likewise, in the experiments that involved a swap between high and low P_i_ concentrations, root fraction developed in the opposite fashion compared to the experimental data (Figs G and H in S2 File, compare with Figs 5 and 6). Sugar dynamics appeared remarkably similar between the two models, except for the fact that Thornley's simulations lacked the daily oscillations observed in our model as a consequence of the day-and-night cycle (Fig I in S2 File, compare with Fig D in S2 File).

### Global quantitative assessment of the two models

**Fig 10:**
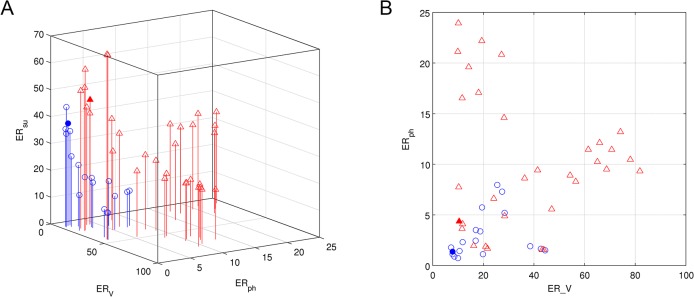
Comparison of models with Pareto fronts. (A) Pareto front of our model (open blue circles) and Thornley’s one (open red triangles). The filled circle and triangle represent the value of (*RE*_*V*_, *RE*_*ph*_, *RE*_*su*_) for the parameter set used in this paper for our model (see S1 and S2 Tables) and Thornley’s (see S3 Table) respectively (obtained with the Nelder-Mead method). (B) Projection of the Pareto fronts on the plan (*RE*_*V*_, *RE*_*ph*_).

In order to compare the two models in a more quantitative way, Pareto fronts for plant volume (*RE*_*V*_), total P_i_ content (*RE*_*ph*_), and soluble sugar content (*RE*_*su*_) were calculated (see Materials and Methods for a detailed description). A Pareto front assesses the global deviation of a set of simulated data points relative to the corresponding experimental data set [82]. A three-dimensional representation of the Pareto front for both models is shown in Fig 10A. Fig 10B shows the corresponding two-dimensional representation featuring only *RE*_*V*_ and *RE*_*ph*_ whereas *RE*_*su*_ was omitted, since it is difficult to compare between the two models because of the fundamentally different submodels for sugar dynamics. While the Nelder-Mead point indicated a good performance for both models (filled circle and triangle in Fig 10), the Pareto front calculated from our model with the genetic algorithm (blue circles in Fig 10) was consistently closer to the origin than Thornley’s (red triangles in Fig 10), indicating that our model provides generally better results for all optimal parameter sets considered. Notably, all optimized parameter sets for Thornley’s model predicted an inverse root fraction relative to the observed data in the time frame of the experiment.

### The dynamics of balanced growth

Upon closer inspection, we noticed that the RF exhibited an initial unexpected inversion also in the experimental data set, although only for a short transitional period of 4 days, after which the RF developed in a way consistent with balanced growth, i.e. low P_i_ supply (10 μM) caused the RF to increase more than high P_i_ supply (100 μM) (Fig 4C). The initial inversion was statistically significant (p = 0.04585 with Wilcoxon’s test; 5 replicates), indicating that it might be informative for the understanding of relative growth dynamics of the shoot and the root. Interestingly, our model also predicted an initial inversion of RF, although to a lesser extent, but with the same timing (switch after approximately 3.4 days). This finding prompted us to evaluate whether the aberrant behavior of the RF in Thornley’s model could represent an extended duration of this transient inversion. Indeed, simulations over longer time periods revealed a switch at a late time point after 45.8 days from the onset of the treatment (see Supporting Information) if we took *f*_*P*_ ≠ 0. Thus, it appears that the two models differ not in their principle behavior, but in their dynamics. Mathematical analysis (see Supporting Information) of the behaviour of the two models showed that all models that have a similar structure with two compartments (shoot and root), at least six state variables (in the case of Thornley *W*_*s*_, *W*_*r*_, *C*_*s*_, *C*_*r*_, *P*_*s*_, *P*_*r*_), and a root growth rate which represents an increasing function of the root P_i_ concentration, inevitably produce an initial inversion that may return to balanced growth after a variable amount of time. Interestingly, tests on teleonomic models revealed that they can in principle not reproduce the initial inversion observed in our experimental data set (data not shown).

## Discussion

### Adaptive growth responses of plants

Plants as sessile organisms have evolved numerous adaptive strategies to cope with environmental stresses such as heat, drought, shading, and nutrient limitation. Some physiological adaptations involve specific gene expression programs, as, for example, the responses to drought and P starvation [83, 84]. In addition, plants can respond to environmental stresses with pronounced morphogenetic responses such as etiolation and skotomorphogenesis [85], shade avoidance syndrome [86], and cluster root formation [87]. Less dramatic, but of general importance in land plants, are changes in the relative ratio between the amount of shoot and root tissues, which represent part of an adaptive program that compensates limiting light or nutrient supply [88]. Based an a rich body of experimental evidence from many plant species, it is generally assumed that in plants, resources are directed preferentially to the organ that provides the limiting service, i.e. low nutrient supply results in preferential root growth, whereas low light levels promote preferential growth of the shoot [2, 89]. This phenomenon has been termed 'balanced growth' hypothesis or 'functional equilibrium' hypothesis, and it has been the target of numerous experimental and theoretical studies [9, 90, 91].

### Concepts of resource partitioning in plants

A global understanding of resource partitioning requires an integrated view that includes all plant parts and all involved mechanisms. This requires mathematical approaches to integrate all components relevant for the phenomenon. Conceptually, resource partitioning could be controlled either by one or few central partitioning functions, or by many local (decentralized) mechanism that act largely independently of each other, but interdepend as elements of a network and that determine partitioning as an emergent property of the system as a whole. Reflecting these two opposing views, mathematical models of partitioning can be constructed "top down" with few components, or with a "bottom up" approach that considers all the relevant physiological mechanisms involved in partitioning. The advantage of the first approach is that it is relatively simple and contains a limited number of equations and parameters. The advantage of the second one is that it considers more physiological processes, which therefore can by critically examined by the model. However, both approaches also have their limitations. A model can only address the role of processes that it features, hence, simpler models can address only a limited number of questions, whereas, on the other side, more complete models are more versatile, but also complex and computationally expensive. In order to analyze the mechanisms involved in the balanced growth of petunia, we decided to opt for a strategy of the second type ("bottom up"), but with a limited number of involved processes. As a central element, we introduced a day-and-night cycle. This decision is based on the fact that plants have evolved to live in a rhythmic environment, and most physiological processes relevant for partitioning exhibit a rhythmic behavior [92].

### A mathematical model to address the dynamics of shoot and root growth

In order to understand how the physiological activities of the root and the shoot influence each other, and to address how they impact on growth of the plant as a whole, we took a combined experimental and modeling approach. Our plant model consists of two main compartments, the photosynthetic shoot, and the root system whose activity in the model is restricted to the acquisition of P_i_ as a representative for all mineral nutrients. Hence, the relevant environmental factors in our model are light and P_i_ supply, and the question is how they influence the relative growth dynamics in the shoot and the root.

Our model is mechanistic in the sense that it reflects physiological mechanisms in the plant as closely as possible, without going into unnecessary detail. Shoot and root activities are interdependent and are embedded in a realistic environment with a day-and-night cycle. We have used concepts from previous growth models [15, 22, 24, 50], and have introduced the following additional features which are indispensable to understand the dynamics of growth and partitioning in plants under natural conditions: i) a transient carbohydrate storage pool (corresponding to starch), ii) an internal oscillator (corresponding to the circadian clock), iii) metabolic costs for P_i_ uptake, transport of P_i_ and sugars, and for growth, iv) a phloem transport mechanism that is based on mass flow, v) a phloem-based recycling mechanism for P_i_ between the shoot and the root, and, vi) the dependence of leaf thickness on light intensity. A central tenet of our model is the assumption that all regulatory mechanisms act locally, i.e. there is no central decision-taking entity that controls partitioning. Resource partitioning in the model is thus the emerging outcome of photosynthesis, P_i_ uptake, sugar and P_i_ transport, and of their local use in growth of new volume and in respiration.

The introduction of the circadian clock into our model was triggered by our observation that a virtual plant without an endogenous synchronizing mechanism was at risk of misusing its carbon stocks during the night, leading either to left-over starch in the morning, or to starch depletion and death of the plant during the course of the night (data not shown), whereas real plants exploit their carbohydrate stocks very efficiently [43]. Indeed, plants have mechanisms to adapt starch accumulation and degradation to photoperiod and time of the day [93], a mechanism that requires the circadian clock for coordination of metabolism and environment [43]. Consistent with this finding, mutant analysis in *A*. *thaliana* has revealed that the circadian clock promotes growth and productivity [60], thereby conferring a significant selective advantage to plants with a clock. Furthermore, approximately 8000 genes in *A*. *thaliana* are regulated rythmically [92], suggesting that many metabolic processes are under the control of the circadian clock. Accordingly, our modeling approach indicated that the clock is essential to grasp the dynamics of plant growth in a rythmic environment.

### Validation and predictions of the model

We have generated two independent experimental data sets, one for parameter estimation (experiment 1; experiment 2, treatments A and B), and one for validation of the model (experiment 2, treatments C and D; experiment 3). This procedure is a central requirement for rigorous testing of a mathematical model. The results from model validation were encouraging, indicating that the model is based on plausible principles. Furthermore, the adaptive behavior of the model reflected natural plant growth remarkably well. Hence, the structure of the model as a whole is able to grasp the dynamics of resource allocation and differential growth in plants. However, under extreme growth conditions, for example when plants grew at very low P_i_ levels (1 μM), the simulations deviated significantly from the experimental data (Fig 7). This deviation may be due to emergency programs that allow plants to survive and to grow under minimal P_i_ conditions that would lead to growth arrest or death of the virtual plant. Obviously, our model does not reflect the full adaptive potential of real plants like, for example, the P_i_ starvation response [94]. However, our goal is to simulate plant growth within physiological limits relevant for agriculture and not under extreme conditions.

### Evaluation of critical components of the model

In order to test the roles of the central components of the model, individual submodels were either removed or modified to reveal their role in the global behaviour of the model. Removal of either the starch reserves or of the clock resulted in the death of the plant due to sugar depletion during the night period (data not shown). This emphasizes the drastic evolutionary constriction resulting from the rhythmic environment onto plants. We conclude that starch reserves and a circadian oscillator are indespensable for modeling of plant growth in a rythmic environment. More subtle changes in photoperiod (Figs H-J in S1 File) revealed another important feature of plant metabolism: The circadian clock has limited flexibility in its ability to adapt to different photoperiod lengths, a fact that may be related to the molecular components of the clock [95]. It is interesting to note that similar effects on growth and survival have been observed when *Arabidopsis* plants were grown with inappropriate photoperiods, either due to mutations in components of the clock, or due to manipulation of photoperiod [60], thus documenting the central importance of the clock for plant fitness and survival. Interestingly, recent evidence documented an intimate association of the clock with carbohydrate metabolism [96], consistent with the pivotal role of the clock in coordinating the switch between phototrophic metabolism during the day, and heterotrophic metabolism during the night.

As a central element of the model, we addressed the importance of phloem transport, which could potentially represent a limiting factor in plant partitioning. Indeed, increasing or decreasing transport resistance of the phloem had a strong influence, in particular on growth of the heterotrophic root, which represents the major sink for carbohydrate resources in vegetative plants, and therefore depends strongly on efficient carbohydrate supply (Fig 8). In this context it is interesting to note that pathogens that reside in the phloem and interfere with phloem transport lead to comparable negative growth effects that are accompanied with retention of resources in source tissues, and depletion in the sinks [97].

Light and mineral nutrients are the primordial exogenous determinants of plant growth. Hence, we simulated shoot and root growth under conditions of simultaneous sugar and P_i_ shortage in different combinations. Under these conditions, we obtained predictions that are in good agreement with the expected compromise that plants are forced to reach in their respective allocation of resources to the root and the shoot. For example, relative root growth, that was strongly affected by growth reductions under low light conditions, recovered partially when, in addition, P_i_ became limiting (Fig 9). These results show that our model can simultaneously integrate environmental information from light and P_i_ supply and reach balanced growth strategies.

### Comparison of our model with Thornley’s growth model

The model presented here indicates that balanced growth may be an emergent feature of plants. This is an important difference to teleonomic models, in which the balanced growth behavior (or any other desired behavior) is defined as part of the model. In order to evaluate the dynamic behavior of our model relative to previously published models of growth and partitioning, we chose to compare it with a standard growth model described by Thornley [24]. In general, Thornley’s model produced satisfactory results. For example, the simulations of P_i_ content (Fig F in S2 File, panels d,e) and of maximal growth under favorable growth conditions (Fig E in S2 File) were remarkably close to the experimental data. However, despite several attempts with various parameter sets, Thornley’s model never predicted the correct root fraction (RF) within the time frame of our experiments (Fig F in S2 File, panel c). Hence, a central aspect of adaptive plant growth cannot be reproduced with this model. In this context, it should be noted that the parameters of Thornley’s model were fitted under more permissive conditions, namely with relatively large biological intervals $\left(\frac{1}{4}\cdot p_{0},4\cdot p_{0}\right)$ around the initial parameter value *p*_0_ instead of ca. +/- 10% as in the case of our model, and yet, the fitting did not yield a parameter set that led to correct simulations.

In order to address the cause of the deviation in root fraction, we inspected the individual components of Thornley’s model. The good results of the parameter fitting for the submodels of P_i_ uptake (Fig B in S2 File), and for shoot and root growth (Fig E in S2 File), suggest that the inversed RF does not result from these submodels. Alternatively, the aberrant RF in Thornley’s model may result from the transport models that invoke a diffusive mechanism instead of mass flow, which is commonly thought to drive sugar transport in the phloem [45, 98], and P_i_ transport in the xylem (including P_i_ recycling in the phloem). However, the prediction of sugar levels in the shoot and the root were remarkably similar between the two models with largely constant average sugar levels (compare Figs D and I in S2 File), except for the diurnal oscillations in our model. This latter behavior is consistent with the virtually stable levels of sucrose in *A*. *thaliana*, and the rythmic increase of glucose during the course of the day [93].

Considering the multiple differences between the two models, it appears difficult to pinpoint the reason for their divergent behavior. Likely, the characteristics of the predictions represent emergent features, hence their differences cannot easily be traced back to a single causal component of one or the other model. Hence, we decided to carry out a global analysis to compare the behavior of the two models.

### Global comparison of our model with Thornley’s

An important difference between the two models is the number of their parameters. While our model contains 55 parameters, Thornley’s has only 17. How does this affect the behavior of the two models? If our model was built on Thornley’s by adding additional features, then our additional parameters would be expected to improve the quality of the fit due to the additional degrees of freedom. However, due to inherent fundamental differences between the two models, the solutions from his model are not part of the solutions of our model. Importantly, 21 of our parameters were fixed (S1 Table) before global fitting and the others were varied in narrow biological intervals (see S2 Table). This limits the potentially beneficial effect of the additional degrees of freedom brought about by additional parameters, and it may even compromise the results of global fitting because of the additional complexity and of unexpected interactions between the submodels. To compare the two models in a more global and systematic way, and to explore whether Thornley’s model may potentially work better with different parameter sets, a Pareto front was calculated for both models [82] (see Supporting information). This involved the calculation (using a genetic algorithm) of a family of parameter sets by minimizing the relative quadratic error (RE) between simulated and observed values for (1) shoot and root growth, (2) P_i_ concentration, and (3) soluble sugar concentration. For each optimized parameter set, a point in the 3-dimensional space was obtained (the value of these three quadratic errors). The set of all these points is called a Pareto front. It turned out that our front was closer to the origin than Thornley’s, meaning that for all the optimized parameter sets considered, our model performed better than Thornley’s (Fig 10). Interestingly, all the parameter sets of Thornley’s Pareto front produced inversed root fractions, suggesting that Thornley’s model suffers from an inherent limitation that prevents it from reproducing realistic dynamic behavior.

Mathematical analysis revealed that both models produced initial inversions of RF that later turned to a growth behavior consistent with balanced growth. However, the dynamics in the two models were fundamentally different. Our model predicted only short transient inversions with similar dynamics as in the initial inversions observed in the experimental data (Fig A in S3 File), whereas Thornley’s model produced very long inversions (Fig B in S3 File) or even the return to deviant RF after an intermediate phase of balanced growth (Fig C in S3 File).

## Conclusions and Outlook

We describe a combined experimental and modeling approach to examine balanced growth in *Petunia hybrida*. It is based on a mechanistic model that features the core metabolic pathways involved in the generation and distribution of carbohydrate resources, and in nutrient uptake from the soil. Our model involves a day-and-night cycle, starch reserves, and realistic regulatory principles for the conversion of sugars to starch during the day, and for starch degradation during the dark phase. Our functional tests of the model show the necessity of all implicated components, including a circadian clock that is required for the coordination of plant metabolism with the environment. Our model can be used and further developed as a tool for the interpretation of the complex phenotypes of mutants in starch metabolism and in other aspects of primary metabolism and resource partitioning. In addition, such mathematical models will be essential tools for molecular breeders in attempts to manipulate starch production, or other aspects of resource partitioning. Such strategies have been notoriously difficult and often lead to unexpected results, mostly due to the inherent complexity of the underlying pathways and their interactions in multidimensional networks [99]. As stated by Shachar-Hill [100] "The major challenges to success in applying network flux analysis to plant metabolic engineering center on complexity and ignorance". While molecular-genetic studies improve our understanding of the components of metabolic pathways and their individual functions, mathematical modeling is the method of choice to address their interactions in complex networks, and to examine what global properties emerge from such networks.

## Supporting Information

S1 File Model building and parameter estimation. (PDF)

S2 File Comparison with the model of Thornley (1998). (PDF)

S3 File Mathematical analysis of root fraction in Thornley's model. (PDF)

S1 Table Fixed parameters of the mathematical model. (PDF)

S2 Table Fitted parameters of the mathematical model. (PDF)

S3 Table Parameter values of Thornley’s model. (PDF)

S4 Table Original data for Figs 1–7. (XLS)
