# Supplementary material for: Mathematical Modeling of the Dynamics of Shoot-Root Interactions and Resource Partitioning in Plant Growth
Source: PLoS One. 2015 Jul 8;10(7):e0127905. doi: 10.1371/journal.pone.0127905 (PMC4495989; doi:10.1371/journal.pone.0127905)
Supplement: S1 File — (PDF) [file pone.0127905.s001.pdf]

Mathematical modeling of the dynamics of shoot-root interactions and resource partitioning in plant growth

Chrystel Feller, Patrick Favre, Ales Janka, Samuel C. Zeeman,
Jean-Pierre Gabriel, Didier Reinhardt

S1 File

Model building and parameter estimation

Three functions were used to model saturation kinetics and regulatory feedback mechanisms: The Monod function, increasing sigmoids, and decreasing sigmoids (Figure A in S1 File).

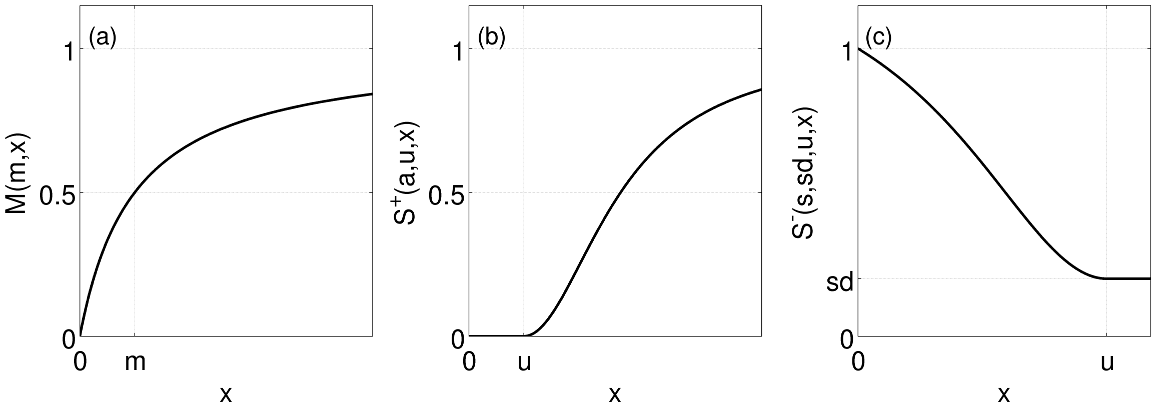

Figure A: Functions used to model regulatory feedbacks and saturation kinetics.
a) Monod function. The parameter m corresponds to 0.5 in ordinate. b) Increasing sigmoid. The parameter u corresponds to the starting point. c) Decreasing sigmoid. The parameters s_d and u correspond respectively to the minimum of the function and the point at which the function reaches s_d.

Photosynthetically active leaf area

Hypotheses 1 and 2 (about leaf thickness and photosynthetically active surface area) were tested by fitting the photoactive leaf surface S^{sh}_{photo} to a dataset for (V^{sh}, S^{sh}_{photo}) with three different light intensities I_{max} (Figure B in S1 File). Photoactive leaf surface was assumed to correspond to the projected leaf area, hence, plants were imaged from above.
The resulting parameters were:

$$th_{min} = 0.026\ cm, th_{max} = 0.084\ cm, S_c = 22.445\ cm^2 \text{and} m_{th} = 592.0194\ \mu mol\ m^{-2}\ s^{-1}$$

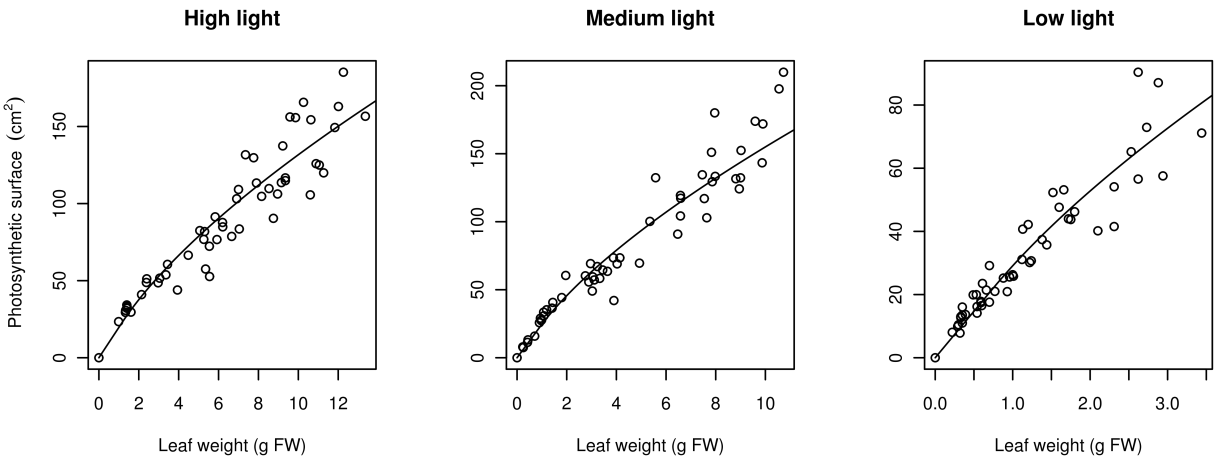

Figure B: Photosynthetically active leaf surface.
Theoretical photosynthetically active leaf surface, based on hypothesis 2 (shading) as a function of leaf volume and light intensity, fitted to the observed projected leaf area.

Phloem tube number and length

We tested hypothesis 4 by fitting the submodel for phloem tube length L:

$$L(V^{pl}(t)) = \ell_1 M_L(V^{pl}(t)) + \ell_2 V^{pl}(t)$$

to experimental data, which resulted in the following parameters:

$$\ell_1 = 8.999\, cm, \ell_2 = 0.321\, cm \text{and} m_L = 0.192\, cm^3.$$

The resulting prediction of the submodel can be found in Figure C in S1 File.

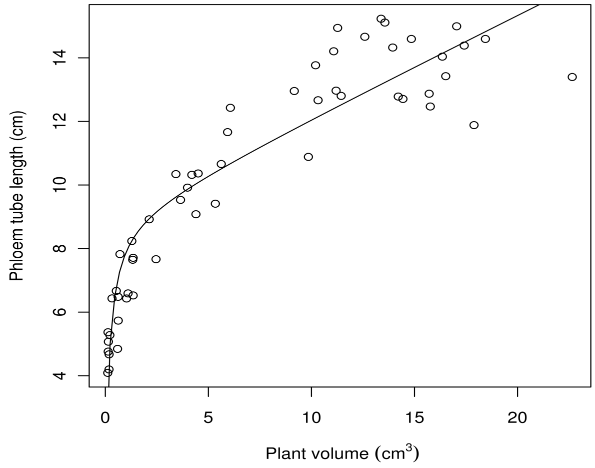

Figure C: Relation of phloem tube length and plant size.
Phloem tube length in the model as a function of plant volume fitted to experimental data.

The submodel for the number of phloem tubes *n* (Hypothesis 5) was validated by fitting its parameters to experimental data:

$n_{min} = 13.028,\ n_{max} = 7494.676,\ a_n = 9677.612\ (cm^3)^2$ and $u_n = 0\ cm^3$, resulting in the prediction of Figure D in S1 File.

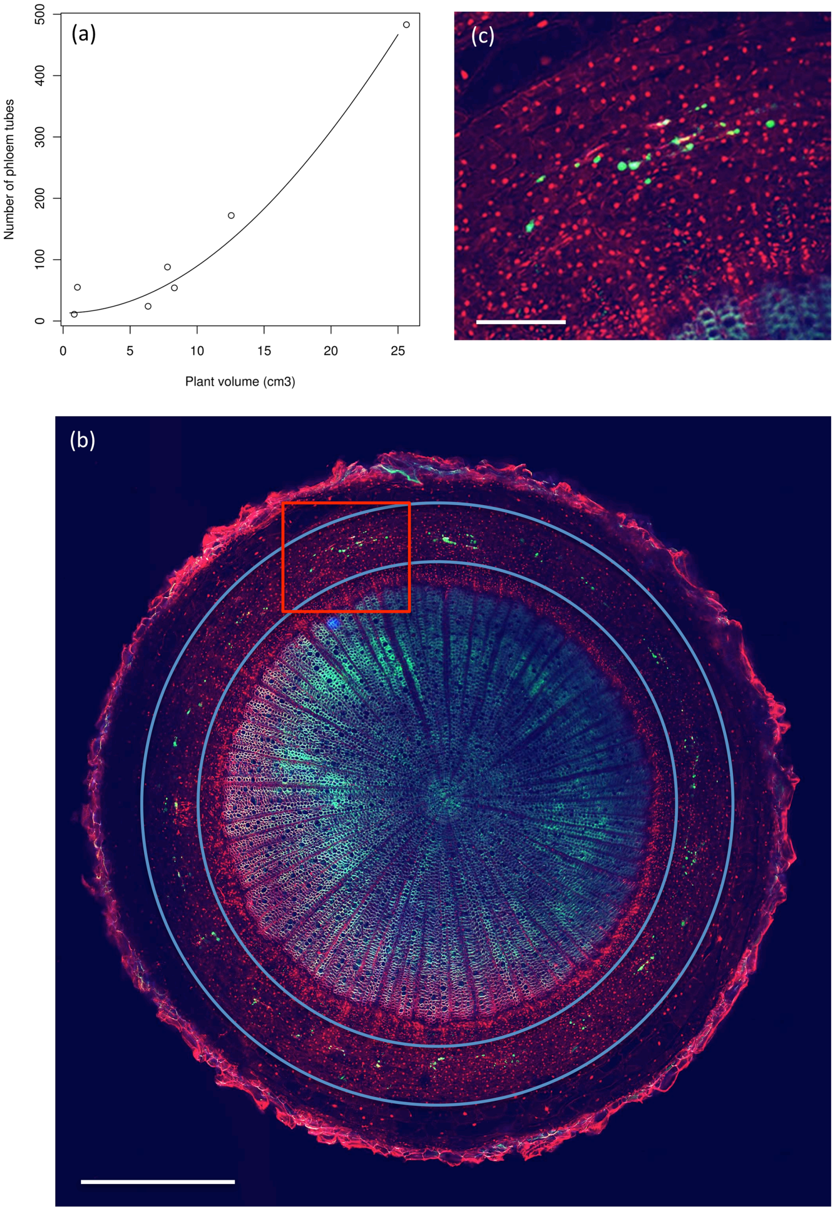

Figure D: Estimation of phloem tube number as a function of plant weight.
(a) Experimental quantification of phloem tube number (circles) and fitted curve as a function of plant weight. (b) Callose staining to reveal the phloem tubes. Green pixels were automatically quantified between the two blue circles. (c) Inset as indicated by the red square in (b); green dots represent phloem tubes. Size bar 1 mm in (b), 200 µm in (c).

Growth parameters and carbohydrate metabolism

The parameters of shoot and root growth were determined by fitting shoot and root weight in time t_i to the model by integrating shoot and root growth to the experimental dataset (experiment 2 A and B) as follows:

$$V^{sh}(t_i) = V^{sh}(t_0) + \int_{t_0}^{ti} g_{max}^{sh}\; M_{g,sh}(V^{sh}(t))\; S_{g,sh,su}^{+}(C_{su}^{sh}(t))\; S_{g,sh,ph}^{+}(C_{ph}^{sh}(t))\; dt$$

$$V^{r}(t_i) = V^{r}(t_0) + \int_{t_0}^{ti} g_{max}^{r}\; M_{g,r}(V^{r}(t))\; S_{g,r,su}^{+}(C_{su}^{r}(t))\; S_{g,r,ph}^{+}(C_{ph}^{r}(t))\; dt$$

The fitted curves are represented in Figure E in S1 File.

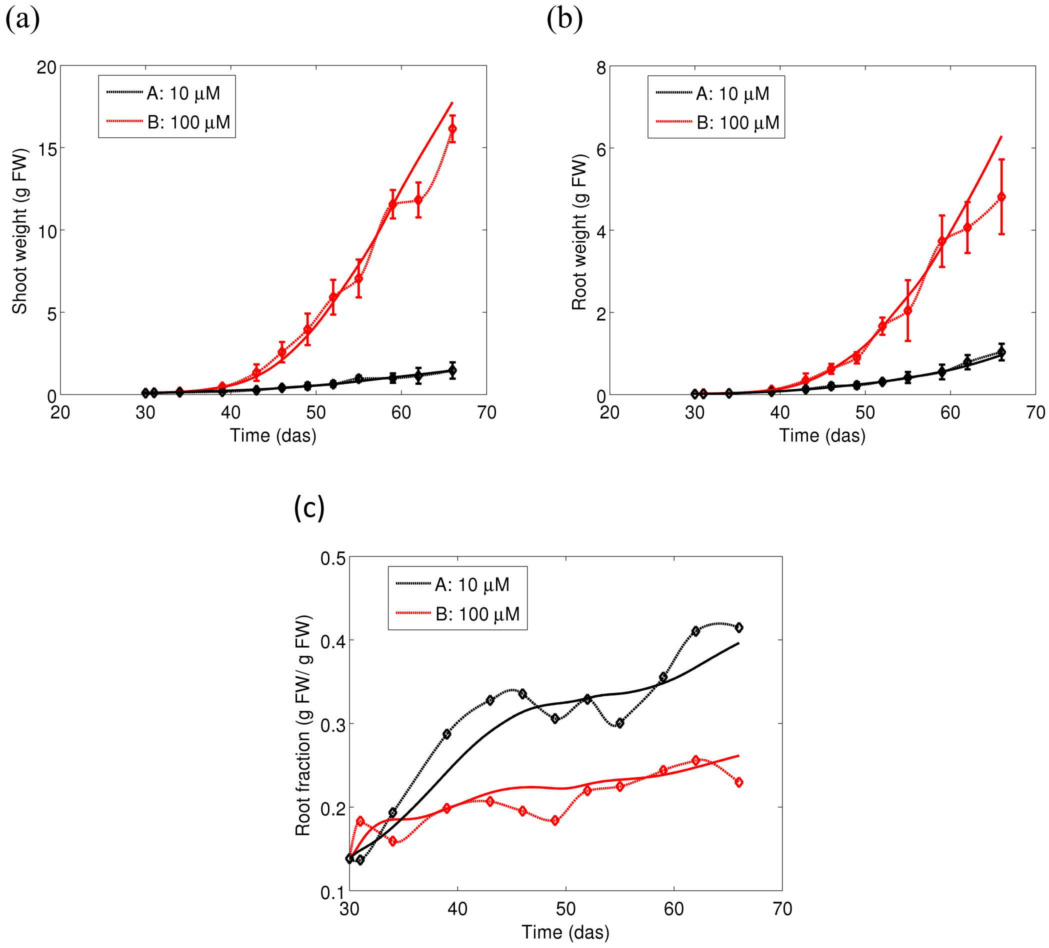

Figure E: Shoot and root weight as a function of phosphate supply.
Simulated shoot (a) and root (b) weight, and root fraction (c) fitted to the observed points for 10 μM (black points) and 100 μM (red points) P_i. Das: days after sawing.

With this procedure, we obtained the following parameters for shoot and root growth:

$$\begin{aligned}
g_{max}^{sh} &= 0.203\, cm^3\, h^{-1}, & m_{g,sh} &= 19.349\, cm^3, \\
g_{max}^{r} &= 0.084\, cm^3\, h^{-1}, & m_{g,r} &= 7.983\, cm^3 \\
C_{su,gr} &= 360.415\, \mu g\, cm^{-3}, & a_{g,su} &= 1.372\, (\mu g\, cm^{-3})^2, \\
C_{ph,gr} &= 69.693\, \mu g\, cm^{-3}, & a_{g,ph} &= 6.304 \cdot 10^4\, (\mu g\, cm^{-3})^2.
\end{aligned}$$

under the assumptions that:

$$C_{su,gr}^{sh} = C_{su,gr}^{r} =: C_{su,gr}, C_{ph,gr}^{sh} = C_{ph,gr}^{r} =: C_{ph,gr}$$
$$a_{g,sh,su} = a_{g,r,su} =: a_{g,su}, a_{g,sh,ph} = a_{g,r,ph} =: a_{g,ph}$$

A schematic representation of photosynthesis and assimilate storage is shown in Figure F in S1 File.

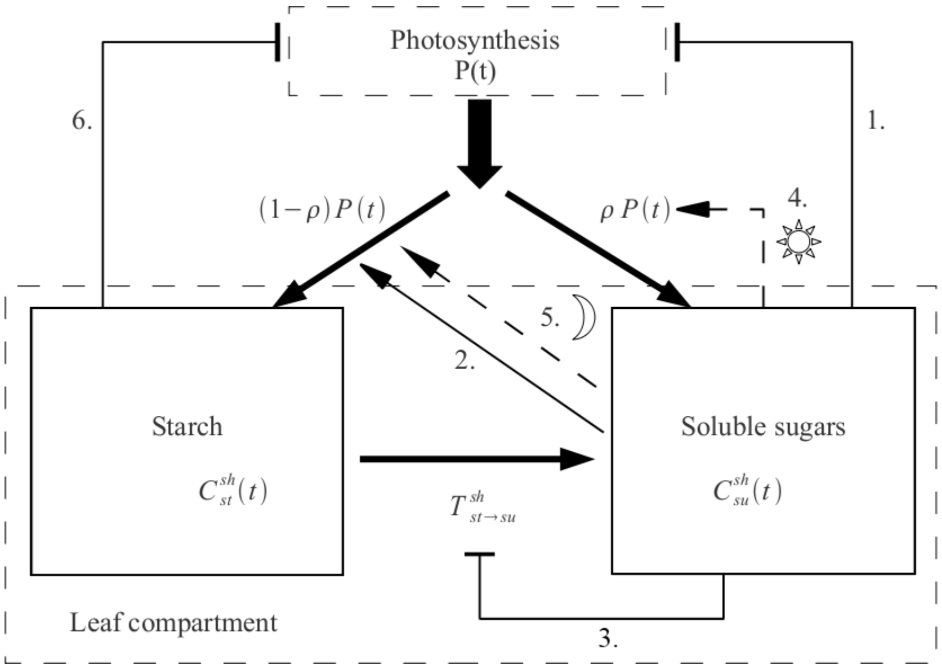

Figure F: Submodel for starch production and degradation in the shoot compartment and regulatory feedbacks. Solid lines represent responses to high carbohydrate concentrations whereas dashed lines represent responses to low carbohydrate levels). Carbohydrate produced by photosynthesis is allocated to the storage pool and the soluble pool according to ρ. During the night, carbohydrate of the storage pool is transferred to the soluble pool with a rate depending on the endogenous circadian oscillator (adjusted to 25 h). This allows to use most of the stored carbohydrate during the night. High soluble carbohydrate concentrations inhibit photosynthesis (feedback 1), promote carbohydrate storage ($(1-\rho)$, feedback 2) and inhibit carbohydrate flux from the storage to the soluble pool (feedback 3), whereas low carbohydrate concentrations during the day increase the value of ρ (feedback 4). In contrast, low carbohydrate levels during the night phase promote carbohydrate storage during the following

day ($(1-\rho)$, feedback 5), thus leading to the build-up of higher levels of reserves during the subsequent photoperiod to avoid carbohydrate depletion during the following night. In addition, high levels of stored carbohydrate inhibit photosynthesis (feedback 6).

Phosphate uptake

The parameters of phosphate (P_i) uptake rate (Hypothesis 6) were estimated indirectly. Total plant P_i quantity Q_{ph}^{pl} at time t_i, obtained by integrating P_i uptake U_{ph} (t_0 is the time after sowing at the beginning of the experiment):

$$Q_{ph}^{pl}(t_i) = \int_{t_0}^{t_i} U_{max}\, S_{max}\, M_U(C_{ph}^{soil}(t))\, M_{as}(V^r(t))\, dt$$

was fitted to the data set of Experiment 2 A and B (with 100 μM and 10 μM) and of experiment 3 (with 300 μM and 1000 μM) (Figure G in S1 File), in order to obtain a better estimation of the parameter m_U. P_i concentration in the soil $C_{ph}^{soil}(t)$ was assumed to remain constant and equal to the supplied concentration (i.e. the P_i concentration in the watering solution). This assumption is justified by the frequent (3x per week) and thorough watering of the substrate (quartz sand), which was assumed to replace the soil solution completely.

Note that the remaining data represented in Figure G in S1 File were not used for parameter fitting, but instead for subsequent comparison with simulations to evaluate the predictive power of the submodel. With this method, we obtained the following parameters:

$$U_{max}S_{max} = 33.297\ \mu g\ cm^{-3}h^{-1},\ m_U = 1.479\cdot 10^4\ \mu g\ l^{-1},\ m_{as} = 0.160\ cm^3.$$

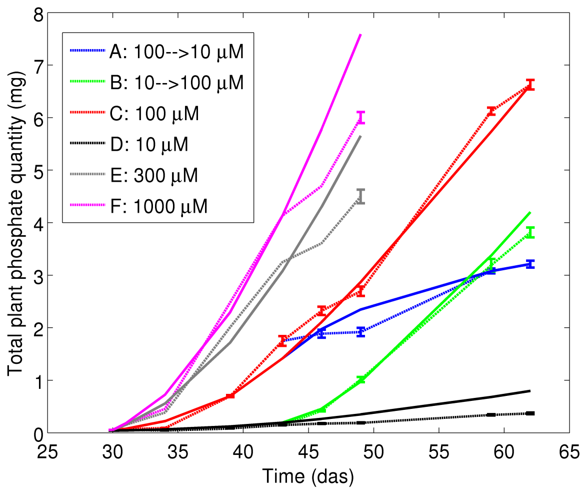

Figure G: Total phosphate pool of plants as a function of phosphate supply.

Modeled total plant P_i quantity fitted to the experimental points for a P_i supply of 10 μM, 100 μM, 300 μM and 1000 μM, and comparison of the predicted values for the two other treatments. Das: days after sawing.

Soluble carbohydrate levels

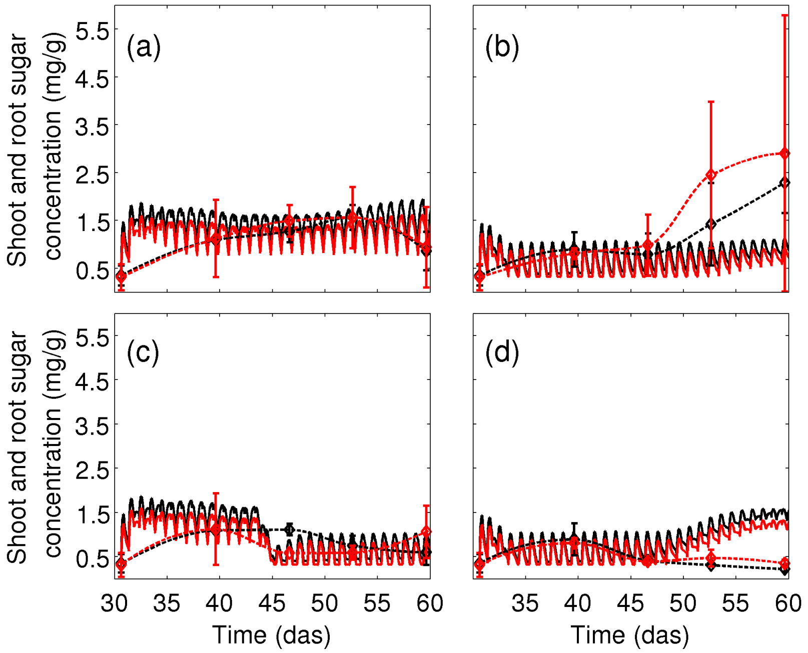

Figure H. Sugar levels as a function of P_i supply in our model.
Total soluble sugar levels (sucrose+glucose+fructose) in the shoot (dashed black lines) and the root (dashed red lines) from experiment 2 (corresponding to Figures 4 to 6), and corresponding predictions of our model (solid lines). Plants were grown at a light level of 316 µM m^{-2} s^{-1} and treated with 10 µM KH_2PO_4 (a), 100 µM KH_2PO_4 (b), or switched from 10 µM to 100 µM KH_2PO_4 (c), or from 100 µM to 10 µM KH_2PO_4 (d). Values represent the mean of five biological replicates with standard deviations. Das: Days after sawing.

Simulation of changes in photoperiod

In order to test the effects of changes in photoperiod onto the model, the day:night setting was changed from 12h:12h to 10h:10h, 14h:14h, and 16h:16h, resulting in a total of 20, 28, and 32 h photoperiod, respectively. Longer photoperiods (14:14 and 16:16) resulted in decreased shoot growth, whereas the shorter photoperiod reduced root growth, resulting in a respective increase and decrease of root fraction (Figure I in S1 File). Shortening of the photoperiod resulted in only partial starch degradation during the night period, whereas extension of the photoperiod resulted in starch depletion during the night (Figure J in S1 File). Soluble sugar levels remained normal at short photoperiod, whereas long photoperiods resulted in sugar depletion during the night, and death of the plant at the 16:16 photoperiod, due to complete exhaustion of sugar levels (Figure K in S1 File).

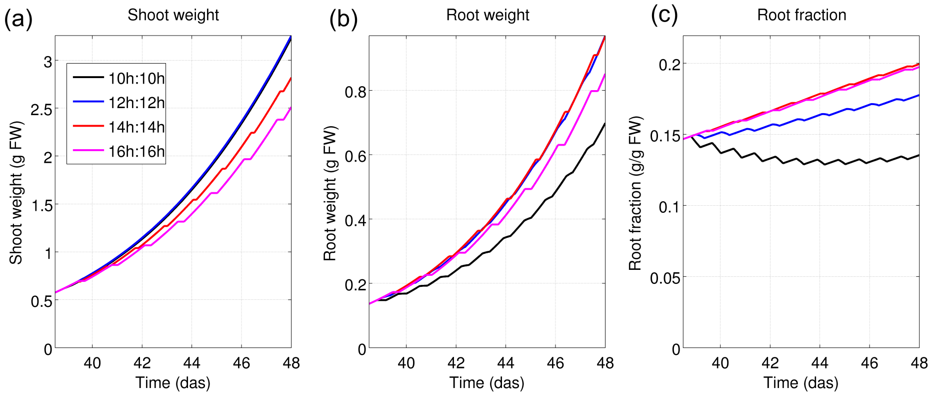

Figure I. Effect of changes in photoperiod from the normal day-and-night cycle (12:12) to 10:10, 14:14, or 16:16 onto shoot growth (a), root growth (b), and root fraction (c). Das: days after sawing.

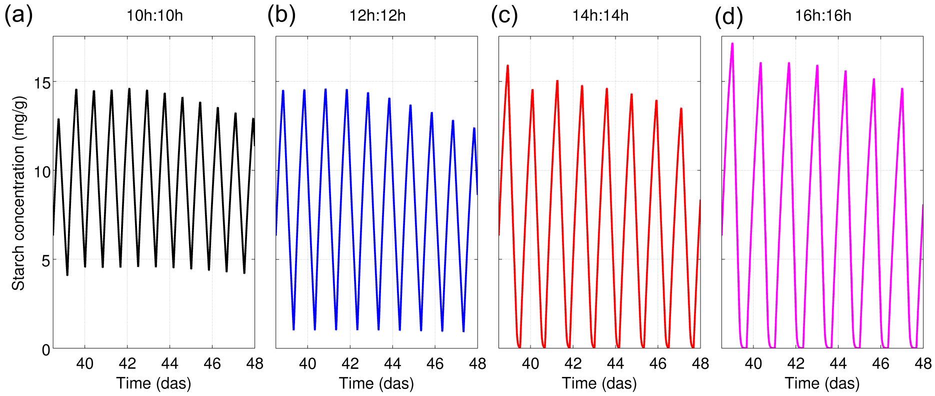

Figure J. Effect of changes in photoperiod from the normal day-and-night cycle (12:12; b) to 10:10 (a), 14:14 (c), or 16:16 (d) on the levels of starch. Das: days after sawing.

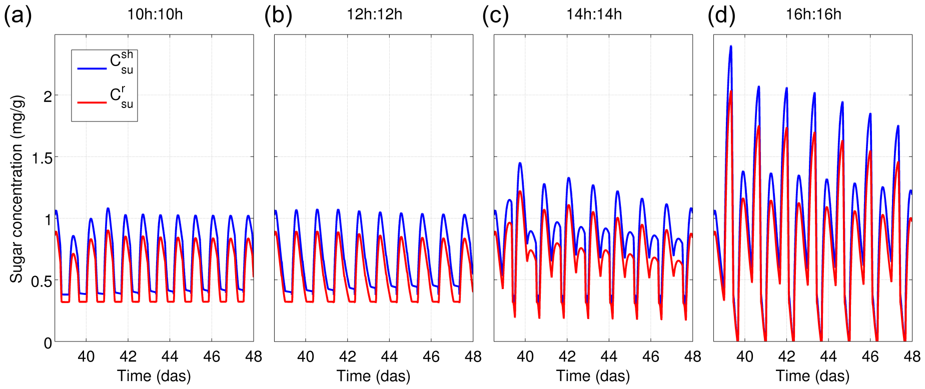

Figure K. Effect of changes in photoperiod from the normal day-and-night cycle (12:12; b) to 10:10 (a), 14:14 (c), or 16:16 (d) on the levels of soluble sugars. Das: days after sawing.

No	Variable	Unit	Exp 1	Exp 2 A and C	Exp2 B and D	Exp3
1	$C_{ph}^{soil}(t)$	$\mu g \quad \ell^{-1}$	28500	950	9500	$95\text{-}1.0418\cdot\ 10^{5}$
2	$Q_{su}^{sh}(t)$	μg	78.820	30.520	30.520	89.075
3	$Q_{su}^{r}(t)$	μg	18.600	4.340	4.340	10.416
4	$Q_{st}^{sh}(t)$	μg	1309.302	506.977	506.977	1479.651
5	$\rho(t)$	$\mu g \quad \mu g^{-1}$	0.5	0.5	0.5	0.5
6	$Q_{ph}^{sh}(t)$	μg	81.297	31.479	31.479	39.753
7	$Q_{ph}^{r}(t)$	μg	58.086	13.553	13.553	15.083
8	$V^{sh}(t)$	cm^{3}	0.131	0.101	0.101	0.296
9	$V^{r}(t)$	cm^{3}	0.0297	0.014	0.014	0.033

Table A The initial states
