# Supplementary material for: Mathematical Modeling of the Dynamics of Shoot-Root Interactions and Resource Partitioning in Plant Growth
Source: PLoS One. 2015 Jul 8;10(7):e0127905. doi: 10.1371/journal.pone.0127905 (PMC4495989; doi:10.1371/journal.pone.0127905)
Supplement: S2 File — (PDF) [file pone.0127905.s002.pdf]

Mathematical modeling of the dynamics of shoot-root interactions and resource partitioning in plant growth

Chrystel Feller, Patrick Favre, Ales Janka, Samuel C. Zeeman,
Jean-Pierre Gabriel, Didier Reinhardt

S2 File

Comparison with the model of Thornley (1998)

Since the growth model of Thornley [1] follows a similar approach as ours and addresses similar questions, we decided to compare the two. This required a number of modifications and adaptations of Thornley's model to make the two comparable. First, we changed the representative mineral nutrient element of the model (nitrogen) to phosphorus (see Table A in S2 File) for the new state variables).

Table A. Plant state variables in Thornley's adapted model

No	Variable	Definition	Unit
1	W_s	shoot fresh weight	g
2	W_r	Root fresh weight	g
3	C_s	Soluble sugar concentration in the shoot compartment	g / g FW
4	C_r	Soluble sugar concentration in the root compartment	g / g FW
5	P_s	P_i concentration in the shoot compartment	g / g FW
6	P_r	P_i concentration in the root compartment	g / g FW

Photosynthetically active leaf area

Thornley's expression for the photosynthetic rate P

$$P = \frac{k_C W_s}{(1 + W_s/K_M)(1 + C_s/J_C)}$$

was modified for the two following reasons: first, Thornley used the same constant K_M for both leaves (in P) and roots (in the P_i uptake rate U); and second, we wanted to isolate the influence of light intensity hidden in the maximal photosynthesis rate k_C.

We thus adapted the submodel of Thornley [1] in the following way:

$$P = k_C \, S_{photo}^{sh} \, \frac{\ell}{k_{CL} + \ell} \, \frac{1}{1 + C_s/J_C}$$

where ℓ is the light intensity, k_{CL} and J_C positive constants and S_{photo}^{sh} the leaf photosynthetic active surface $S_{photo}^{sh} = S_{max} \frac{W_s}{K_{Ms} + W_s}$, with S_{max} and K_{Ms} positive.

The fitting of the parameters of the photosynthetically active leaf surface to experimental data yielded the following parameter set:

$$S_{max} = 251.254 \; cm^2 \text{and} K_{Ms} = 8.225 \; g.$$

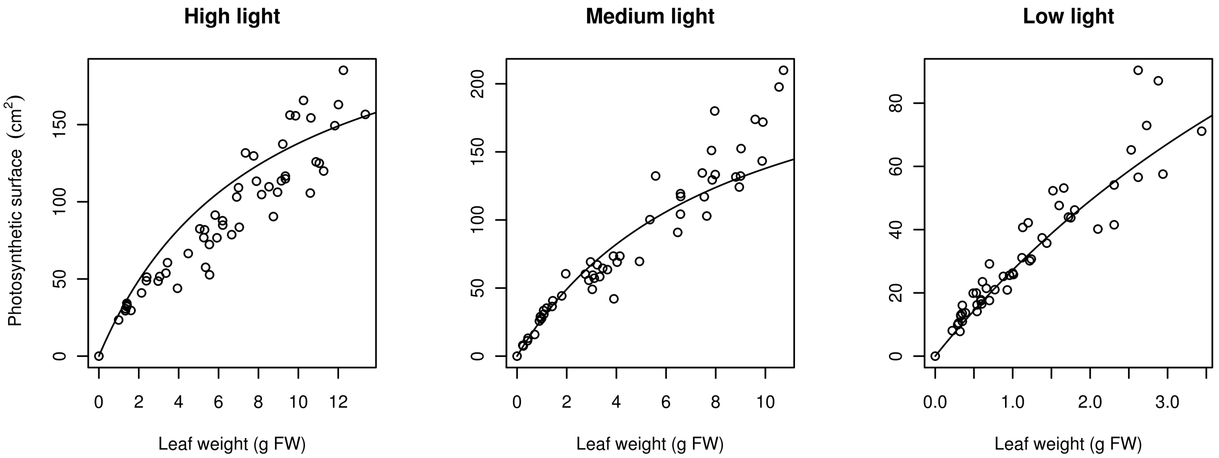

Figure A. Modeling photosynthetically active leaf surface according to Thornley.
Theoretical photosynthetically active leaf surface area is expressed as a function of leaf volume and light intensity fitted to the observed projected leaf area.

Phosphate uptake

The P_i uptake rate was modified as follows. In Thornley's submodel

$$U_P = \frac{k_P W_r}{(1 + W_r/K_M)(1 + P_r/J_P)},$$

K_M was replaced by K_{Mr} and k_P by

$$\frac{k_P}{K_{Mr}} \frac{S}{k_{PR} + S},$$

where S represents P_i concentration in the watering solution and k_{PR} a positive constant rate. Note that P_i concentration in the soil $C_{ph}^{soil}(t)$ was assumed to remain constant and equal to the concentration S.

As for our model, P_i uptake constants were estimated by fitting total plant phosphate quantity Q_{ph}^{pl} in time t_i, (which was obtained by integrating phosphate uptake U_P):

$$Q_{ph}^{pl}(t_i) = Q_{ph}^{pl}(t_0) + \int_{t_0}^{t_i} \frac{k_P}{K_{Mr}} \frac{S}{k_{PR} + S} \frac{W_r(t)}{(1 + W_r(t)/K_{Mr})(1 + P_r(t)/J_P)} dt$$

to the data set of Experiment 2 (treatments A and B with 100 μM and 10 μM) and Experiment 3 (with 300 μM and 1000 μM) (Figure B in S2 File). Again, the remaining data were used not for parameter fitting, but for validation of the model (Figure B in S2 File, treatment 10 μM → 100 μM and 100 μM → 10 μM). With this method, we obtained the following parameters:

$$k_P = 9.495 \cdot 10^{-4}\ g\ d^{-1},\ k_{PR} = 1.802 \cdot 10^{4}\ \mu g\ l^{-1},\ K_{Mr} = 0.192\ g \text{ and } J_P = 2.942 \cdot 10^{-2}\ g\ (g\ FW)^{-1}.$$

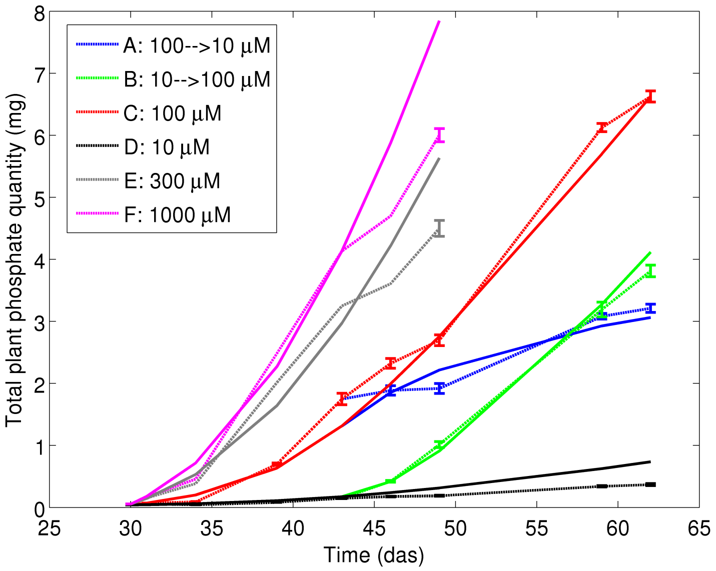

Figure B. Total phosphate pool of plants as a function of phosphate supply modeled according to Thornley.
Simulated total plant P_i quantity fitted to the experimental data for a P_i supply of 10 μM, 100 μM, 300 μM and 1000 μM, and comparison of the predicted values for the remaining treatments (10 μM → 100 μM, 100 μM → 10 μM). Das: Days after sawing.

Thornley modeled carbohydrate and P_i transport with diffusive processes as follows:

$$T_{C:s\to r}=\frac{C_s-C_r}{r_C}\text{ and }T_{P:r\to s}=\frac{P_r-P_s}{r_P}$$

where resistances r_C and r_P are functions of root and shoot weight, respectively:

$$r_C=\rho_C\left(\frac{1}{W_s^q}+\frac{1}{W_r^q}\right)\text{ and }r_P=\rho_P\left(\frac{1}{W_s^q}+\frac{1}{W_r^q}\right)$$

with $q=1$, as a scaling parameter that depends, presumably, on plant architecture, and ρ_C and ρ_P as constants, which were chosen arbitrarily equal to $0.05\,d$ and $1\,d$, respectively.

Growth parameters and carbohydrate metabolism

According to Thornley, shoot and root growth are proportional to carbon and P_i concentration:

$$\frac{dW_s}{dt}=k_G W_s C_s P_s-\frac{k_{lit}W_s}{1+K_{M,lit}/W_s}$$

$$\frac{dW_r}{dt}=k_G W_r C_r P_r-\frac{k_{lit}W_r}{1+K_{M,lit}/W_r}$$

For consistency with our model, we replaced the constant k_G by two different growth rates k_G^s and k_G^r for the shoot and the root compartment, respectively. These constants were estimated by fitting shoot and root weight in time t_i (obtained by integrating shoot and root growth):

$$W^s(t_i)=W^s(t_0)+\int_{t_0}^{t_i}\left(k_G^s W_s C_s P_s-\frac{k_{lit}W_s}{1+K_{M,lit}/W_s}\right)dt$$

$$W^r(t_i)=W^r(t_0)+\int_{t_0}^{t_i}\left(k_G^r W_r C_r P_r-\frac{k_{lit}W_r}{1+K_{M,lit}/W_r}\right)dt$$

to the experimental observations (experiment 2, treatments A and B) with 100 µM and 10 µM, respectively (Figure C in S2 File). This procedure yielded the following parameters for shoot growth:

$$k_G^s=2.699\cdot10^5\ (g\ su/g\ FW)^{-1}\ (g\ Pi/g\ FW)^{-1}\ d^{-1},$$
$$k_G^r=2.7436\cdot10^5\ (g\ su/g\ FW)^{-1}\ (g\ Pi/g\ FW)^{-1}\ d^{-1},$$
$$k_{lit}=8.557\cdot10^{-3}\ d^{-1}$$
$$K_{M,lit}=4.744\cdot10^1\ g.$$

Based on the results shown in Figure C in S2 File, it was concluded that Thornley's submodel for shoot and root growth can predict the data only at early stages when the plant is younger than 55 days.

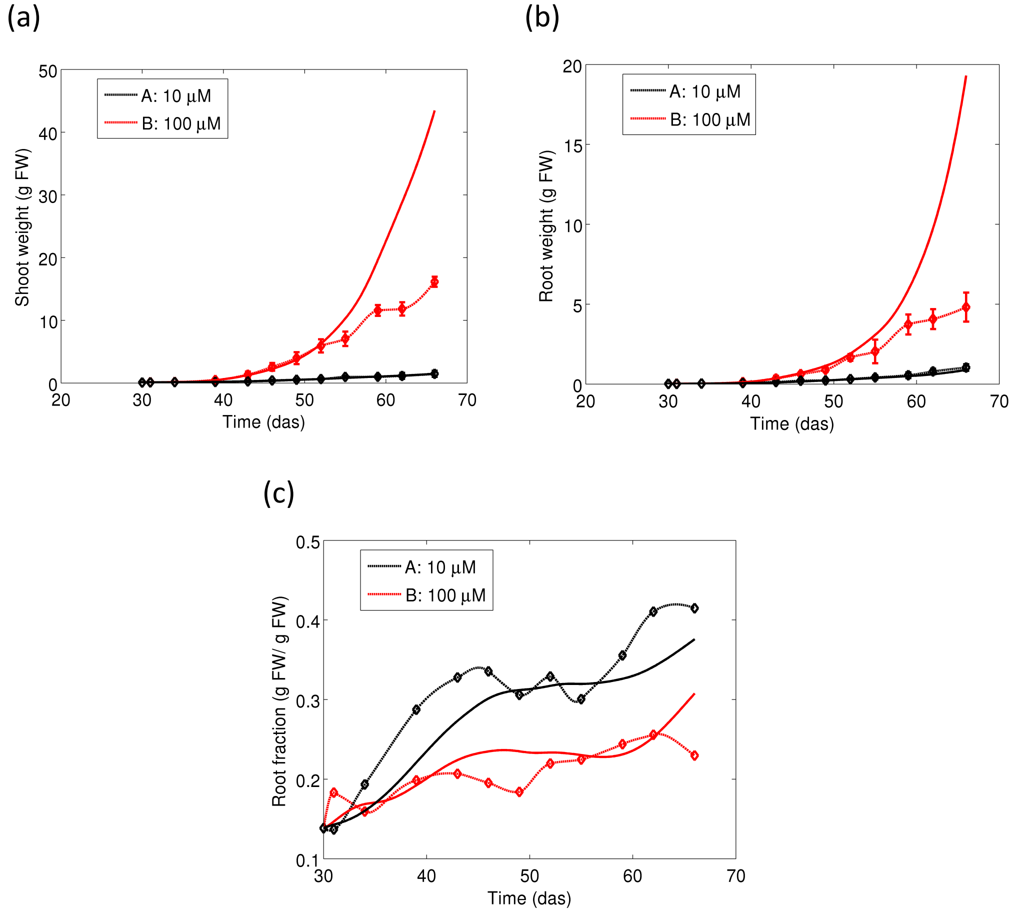

Figure C. Shoot and root weight as a function of phosphate supply modeled according to Thornley.

Theoretical curves obtained by fitting simulated shoot weight (a), root weight (b), and root fraction (c) to the experimental data for 10 µM P_i (black points) and 100 µM P_i (red points). Das: Days after sawing.

The fitted parameters (S3 Table) provided a satisfactory match between simulations and experimental data (Figures D and E in S2 File, S3 Table). However, for the root fraction (RF) the simulations behaved opposite to the observations i.e. the model prioritized shoot growth when P_i supply was low and root growth when P_i supply was high (Figure E in S2 File, panel c).

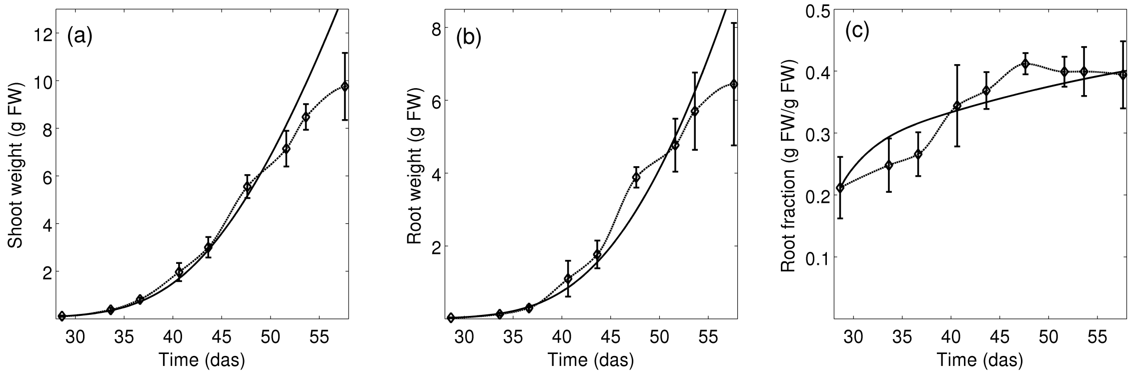

Figure D. Parameter fitting for Thornley's model at optimal growth conditions.
Plants were grown at high light levels (595 µmol $m^{-2}s^{-1}$) and a high P_i concentration in the soil (300 µM). Simulations (continuous line) and experimental data (Experiment 1; dashed line) are shown for shoot growth (a), root growth (b), and the relative root fraction (c). Das: Days after sawing.

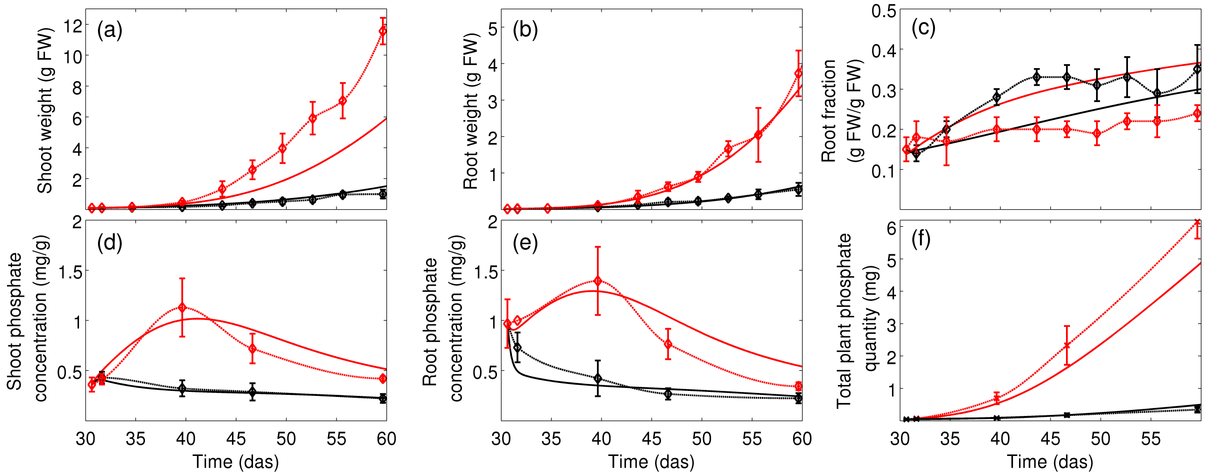

Figure E. Parameter fitting for Thornley's model under two different phosphate levels. Plants were grown at an intermediate light level (316 µmol $m^{-2}s^{-1}$) and at two P_i regimes representing limiting conditions (10 µM, black curves) and intermediate conditions (100 µM, grey curves). Simulations (continuous lines) and experimental data (Experiment 2; treatments A and B; dashed lines) are shown for shoot weight (a), root weight (b), root fraction (c), P_i levels in the shoot (d) and in the root (e), and total P_i in the entire plant (f). Das: Days after sawing.

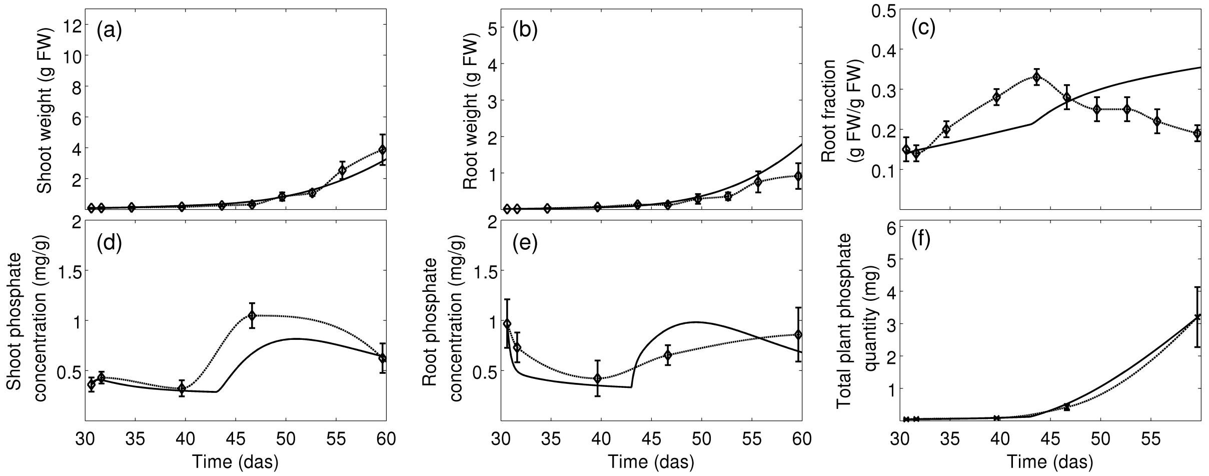

Figure F. Validation of Thornley's model and evaluation of adaptive potential in shoot and root growth. Plants were first grown at low P_i levels (10 µM), followed by a switch to 100 µM after two weeks. Simulations (continuous lines) and experimental data (Experiment 2, treatment C; dashed lines) are shown for shoot weight (a) and root weight (b), root fraction (c), P_i levels in shoot (d) and in root (e) and total P_i in plants (f). Das: Days after sawing.

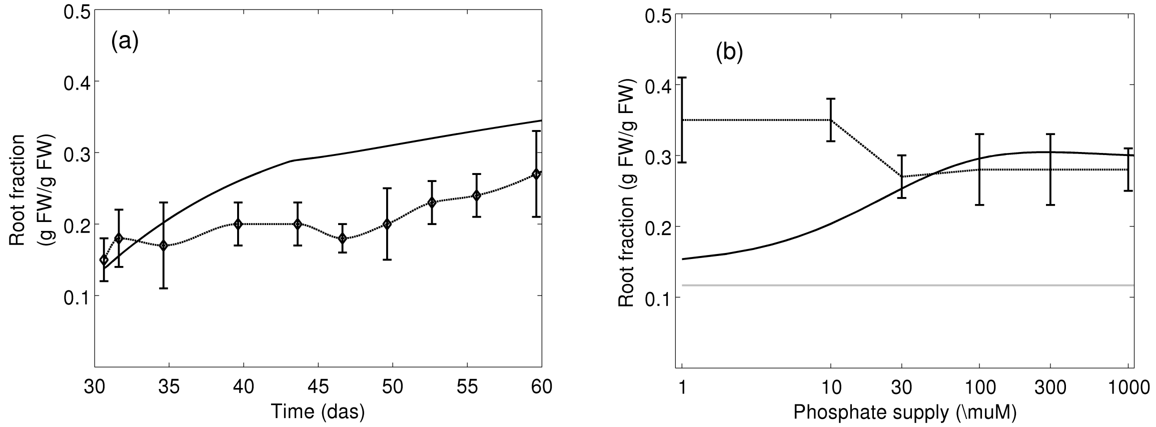

Figure G. Validation of Thornley's model.

(a) Evaluation of adaptive potential in shoot and root growth (experimental design as in Figure F in S2 File), but with reverse switch of P_i solutions). Plants were first grown at high P_i levels (100 µM), followed by a switch to 10 µM after two weeks. Simulations (continuous lines) and experimental data (Experiment 2, treatment D; dashed lines) are shown for the root fraction.

(b) Evaluation of the adaptive potential of plants to a range of different P_i concentrations between 1 µM and 1 mM. Simulations (continuous line; adapted Thornley's model) and experimental data (experiment 3; dashed line) are shown for RF for plants grown under a light intensity of 372 µmol $m^{-2}s^{-1}$ and at 6 different P_i concentrations in the soil (1, 10, 30, 100, 300, 1000 µM) for two weeks. Das: Days after sawing.

Thornley's model with fitted parameters was then compared to the second dataset for model validation (Experiment 2, treatments C and D; experiment 3). The resulting figures (Figures F and G in S2 File) correspond to Figures 5, 6c and 7c, respectively. For Experiment 2 (treatment D) and Experiment 3, only the RF is shown.

In order to understand why Thornley's model produced inverted RF, the results of the individual submodels were inspected. The total quantity of P_i in the plant, and in the two compartments (Figures E and F in S2 File) were in good agreement with the experimental data. Since the parameters were chosen such that the submodel for shoot and root growth matched the data, it is conceivable that the problem lies in carbohydrate transport. As mentioned by Minchin [2], Thornley's submodel for the transport of sugar in the phloem is not based on accepted physiological principles (diffusion instead of mass flow). Alternatively, an oversimplified photosynthesis submodel or the omission of a day-night cycle may cause the model to give these results.

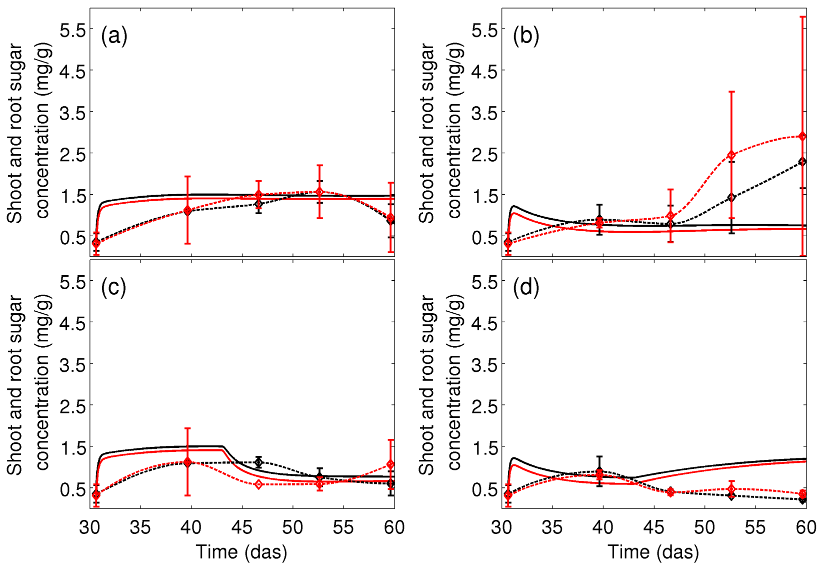

Figure H. Sugar levels as a function of P_i supply in Thornley's model.

Total soluble sugar levels (sucrose+glucose+fructose) in the shoot (dashed black lines) and the root (dashed red lines) from Experiment 2 (corresponding to Figures E and F in S2 File, and to Figure G (panel a) in S2 File), and corresponding predictions of Thornley's model (solid lines). Plants were treated with 10 µM KH_2PO_4 (a), 100 µM KH_2PO_4 (b), or switched from 10 µM to 100 µM KH_2PO_4 (c) or from 100 µM to 10 µM KH_2PO_4 (d). Plants were grown at an intermediate light level of 316 µM m^{-2} s^{-1}. Values represent the mean of five biological replicates with standard deviation. Das: Days after sawing.

Supporting References

1. Thornley JHM (1998) Modelling shoot-root relations: the only way forward? Annals of Botany 81: 165-171.
2. Minchin PEH (2007) Mechanistic modelling of carbon partitioning. In: Vos J, Marcelis LFM, Visser PHB, Struik PC, Evers J, editors. Functional-structural plant modelling in crop production. Wageningen: Springer. pp. 113-122. .
