# Supplementary material for: Mathematical Modeling of the Dynamics of Shoot-Root Interactions and Resource Partitioning in Plant Growth
Source: PLoS One. 2015 Jul 8;10(7):e0127905. doi: 10.1371/journal.pone.0127905 (PMC4495989; doi:10.1371/journal.pone.0127905)
Supplement: S3 File — (PDF) [file pone.0127905.s003.pdf]

Mathematical modeling of the dynamics of shoot-root interactions and resource partitioning in plant growth

Chrystel Feller, Patrick Favre, Ales Janka, Samuel C. Zeeman,
Jean-Pierre Gabriel, Didier Reinhardt

S3_File

Mathematical analysis of root fraction in Thornley's model

In order to address the inversions of RF systematically, we analyzed mathematically the behavior of RF in Thornley's and our model as follows.

Proposition: Let $X(t) = \big(W_s(t), W_r(t), C_s(t), C_r(t), P_s(t), P_r(t)\big)$ be the solution of Thornley's model with initial condition $X(t_0) = x_0 \in R^6_{>0}$. Let $R(t) = \frac{W_r(t)}{W_s(t)+W_r(t)}$ be the RF. After a slight increase/decrease of the maximal P_i uptake rate $\sigma_P = \frac{k_P}{K_{Mr}} \frac{S}{k_{PR} + S}$ at time $t_1 \geq t_0$ i.e.:

$$\sigma_P^\delta = \sigma_P + \delta,$$

the RF of the perturbed system $R^\delta(t)$ has the property:

$$R^{\delta}(t) > R(t) \;\; if \;\; \delta > 0$$
$$R^{\delta}(t) < R(t) \;\; if \;\; \delta < 0$$

for t in a sufficiently small neighborhood of t_1.

Proof:

Let J be the right maximal interval of definition of $X(t)$ (one can prove that $J = [t_0, \infty)$). Assume that $t_1 \in J$. Let $X^\delta(t) = (W_s^\delta(t), W_r^\delta(t), C_s^\delta(t), C_r^\delta(t), P_s^\delta(t), P_r^\delta(t))$ be the solution of Thornley's model with perturbed maximal P_i uptake rate $\sigma_P^\delta = \sigma_P + \delta$ and initial condition $X^\delta(t_1) = X(t_1)$. There exists $\epsilon > 0$ such that $X^\delta(t)$ is defined on $[t_1 - \epsilon, t_1 + \epsilon)$. P_i uptake of the perturbated system is given by

$$U^\delta(t) = \frac{\sigma_P^\delta W_r}{\left(1 + \frac{W_r}{K_M}\right)\left(1 + \frac{P_r}{J_P}\right)} = \underbrace{\frac{\sigma_P W_r}{\left(1 + \frac{W_r}{K_M}\right)\left(1 + \frac{P_r}{J_P}\right)}}_{=U(t)} + \delta \, \frac{W_r}{(1 + W_r/K_M)(1 + P_r/J_P)}$$

We then computed the Taylor development of $R^\delta(t)$ around t_1:

$$\frac{dR^\delta(t)}{dt} = \frac{\dot{W}_r^\delta W_s^\delta - \dot{W}_s^\delta W_r^\delta}{(W_r^\delta + W_s^\delta)^2}$$
$$= \frac{W_r^\delta W_s^\delta}{(W_r^\delta + W_s^\delta)^2}\left((k_G^r C_r^\delta P_r^\delta - k_G^s C_s^\delta P_s^\delta) - k_{lit}\left(\frac{W_r^\delta}{K_M + W_r^\delta} - \frac{W_s^\delta}{K_M + W_s^\delta}\right)\right)$$

and thus

$$\frac{dR^\delta}{dt}(t_1) = \frac{dR}{dt}(t_1)$$

as $X^\delta(t_1) = X(t_1)$. Then the second derivative at t_1 was computed:

$$\left.\frac{d^2R^\delta}{dt^2}\right|_{t=t_1} = \frac{W_r(t_1)W_s(t_1)}{(W_r(t_1) + W_s(t_1))^2}\left.\frac{d}{dt}\right|_{t=t_1}\left(k_G^r C_r^\delta P_r^\delta - k_G^s C_s^\delta P_s^\delta\right)$$
$$-\frac{W_r(t_1)W_s(t_1)}{(W_r(t_1) + W_s(t_1))^2}\left.\frac{d}{dt}\right|_{t=t_1} k_{lit}\left(\frac{W_r^\delta}{K_M + W_r^\delta} - \frac{W_s^\delta}{K_M + W_s^\delta}\right)$$
$$+\left((k_G^r C_r(t_1)P_r(t_1) - k_G^s C_s(t_1)P_s(t_1)) - k_{lit}\left(\frac{W_r(t_1)}{K_M + W_r(t_1)} - \frac{W_s(t_1)}{K_M + W_s(t_1)}\right)\right)\left.\frac{d}{dt}\right|_{t=t_1}\frac{W_r^\delta W_s^\delta}{(W_r^\delta + W_s^\delta)^2}$$
$$= \frac{W_r(t_1)W_s(t_1)}{(W_r(t_1) + W_s(t_1))^2}\left(k_G^r \underbrace{\dot{C}_r^\delta(t_1)}_{=\dot{C}_r(t_1)} P_r(t_1) + k_G^r C_r(t_1)\dot{P}_r^\delta(\mathrm{t}_1)\right.$$
$$\left.-k_G^s \underbrace{\dot{C}_s^\delta(t_1)}_{=\dot{C}_s(t_1)} P_s(t_1) + k_G^s C_s(t_1)\underbrace{\dot{P}_s^\delta(t_1)}_{=\dot{P}_s(t_1)}\right) - \frac{W_r(t_1)W_s(t_1)}{(W_r(t_1) + W_s(t_1))^2}\left.\frac{d}{dt}\right|_{t=t_1} k_{lit}\left(\frac{W_r}{K_M + W_r} - \frac{W_s}{K_M + W_s}\right)$$
$$+\left((k_G^r C_r(t_1)P_r(t_1) - k_G^s C_s(t_1)P_s(t_1)) - k_{lit}\left(\frac{W_r(t_1)}{K_M + W_r(t_1)} - \frac{W_s(t_1)}{K_M + W_s(t_1)}\right)\right)\left.\frac{d}{dt}\right|_{t=t_1}\frac{W_r^\delta W_s^\delta}{(W_r^\delta + W_s^\delta)^2}$$

using $X^\delta(t_1) = X(t_1)$ and the fact that the concentrations $C_s^\delta(t)$, $C_r^\delta(t)$ and $P_s^\delta(t)$ have derivatives in t_1 equal to the derivative of the corresponding component of $X(t)$. We can now express $\left.\frac{d^2R^\delta}{dt^2}\right|_{t=t_1}$ in term of the derivative of $\frac{dR}{dt}$ in t_1:

$$\left.\frac{d^2R^\delta}{dt^2}\right|_{t=t_1} = \left.\frac{d^2R}{dt^2}\right|_{t=t_1} + \frac{W_r(t_1)W_s(t_1)}{(W_r(t_1) + W_s(t_1))^2}\frac{k_G^r\, C_r(t_1)\, W_r(t_1)}{(W_r(t_1))^2(1 + W_r(t_1)/K_M)(1 + P_r(t_1)/J_P)}\,\delta$$
$$=: \left.\frac{d^2R}{dt^2}\right|_{t=t_1} + \alpha\,\delta$$

where we use

$$\dot{P}_r^\delta(\mathrm{t}_1) = \frac{\frac{d}{dt}Q_P^{\delta,r}|_{t=t_1}}{(W_r^\delta(t_1))^2} - P_r^\delta(t_1)\frac{\dot{W}_r^\delta(t_1)}{W_r^\delta(t_1)}$$

$$= \dot{P}_r(t_1) + \delta \frac{W_r(t_1)}{(W_r(t_1))^2(1 + W_r(t_1)/K_M)(1 + P_r(t_1)/J_P)}$$

We have, for $t \geq t_1$:

$$R^{\delta}(t) = R(t) + \frac{\alpha}{2}\delta(t - t_1)^2 + o((t - t_1)^2)$$

where o is the Landau-Symbol i.e. we write $f(h) = \mathrm{o(g(h))}$ for $h \to 0$ if $\lim_{h\to 0}\frac{f(h)}{g(h)} = 0$.

It means that for $t \geq t_1$ in a small neighborhood of t_1, $R^{\delta}(t)$ is larger than $R(t)$ when $\delta > 0$ (as $\alpha > 0$), that is when P_i uptake increases.

This mathematical analysis shows that Thornley's model inevitably produces an inversion of the RF, in which RF at low and high P_i supply develops first in the wrong direction (opposite to the balanced growth hypothesis). The same holds for our model with a similar proof (not shown). Hence, both models predict an inversion of RF at the onset of the experiment. The difference between the two models is the time point at which the RF returns to balanced growth (i.e. the crossing time between the lines representing the two treatments), that is the time $t^* > t_1$ at which the RT of the perturbed $R^{\delta}(t)$ (with $\delta > 0$) becomes equal to $R(t)$ and then smaller than $R(t)$ when $t > t^*$.
We observed in our experimental data that plants indeed exhibited an inverted behavior of the RF until approximately 4 days, and a similar development is predicted by our model (see Figure A in S3_File), whereas Thornley's model predicted more than 45 days (Figures B in S3_File) or even two crossing times and an inverted asymptotic behavior of RF (see Figure C in S3_File) with another parameter set in the Pareto front of Thornley's model. This behavior was never observed with any of the parameter sets of the Pareto front of our model. All of them had a crossing time less than 20 days and none of them crossed a second time (at least until 500 days).

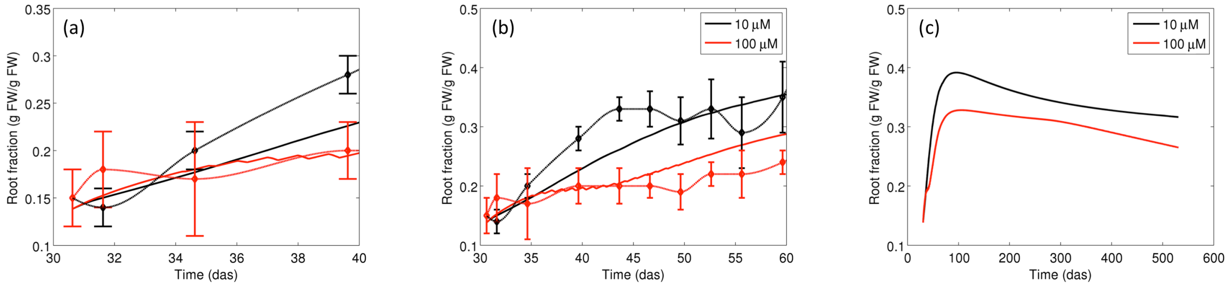

Figure A. Dynamic behavior of root fraction in our model.
RF was modeled during the first 10 days (a), 30 days (b), and 500 days (c) after the start of Experiment 2 (treatments A and B). Experimental data are provided with standard deviations (compare with Figure 4). Das: Days after sawing.

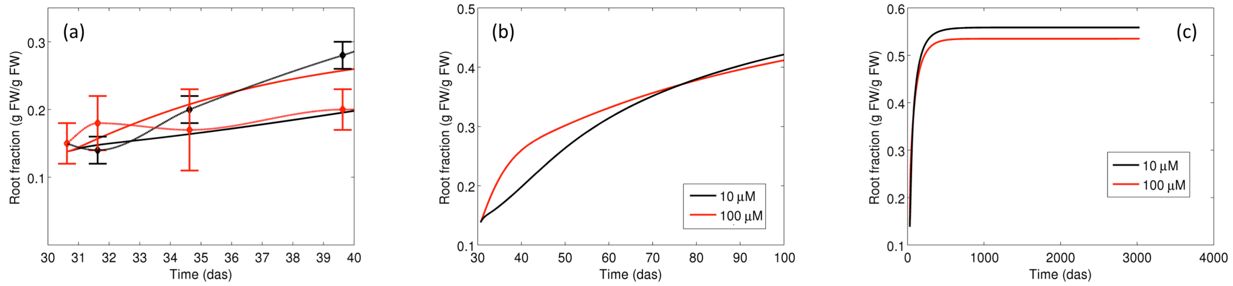

Figure B. Dynamic behavior of root fraction in Thornley's model.
RF was modeled during the first 10 days (a), 70 days (b), and 3000 days (c) after the start of the experiment. Experimental data are provided with standard deviations in (a) (compare with Figure A in S3_File and with Figure 4). Das: Days after sawing.

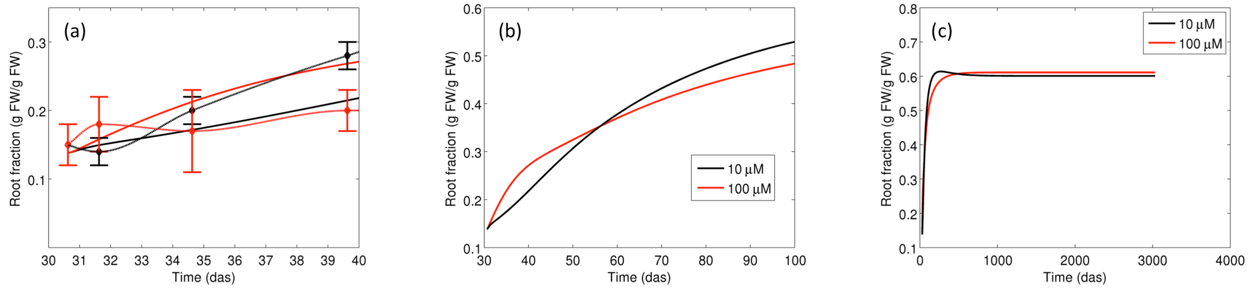

Figure C. Dynamic behavior of root fraction in Thornley's model.
RF was modeled during the first 10 days (a), 70 days (b), and 3000 days (c) with a different parameter set of the same Pareto front for Thornley's model (compare with Figures A and B in S3_File). Das: Days after sawing.
