# Supplementary material for: Mathematical Modeling of the Dynamics of Shoot-Root Interactions and Resource Partitioning in Plant Growth
Source: PLoS One. 2015 Jul 8;10(7):e0127905. doi: 10.1371/journal.pone.0127905 (PMC4495989; doi:10.1371/journal.pone.0127905)
Supplement: S1 Table — (PDF) [file pone.0127905.s004.pdf]

Table S1 Fixed parameters of the mathematical model

Category	Parameter	Value	Units	Source
Leaf thickness	th_{max}	0.084	cm	This study
	th_{min}	0.026	cm	This study
	m_{th}	592.0194	μ mol m^{-2} s^{-1}	This study
Critical surface	S_c	22.445	cm^2	This study
Phloem tube length	ℓ_1	8.999	cm	This study
	ℓ_2	0.321	cm	This study
	m_L	0.192	cm^3	This study
Phloem tube number	n_{max}	7494.676	dimension-less	This study
	n_{min}	13.028	dimension-less	This study
	a_n	9677.612	(cm^3)^2	This study
	u_n	0	cm^3	This study
Phloem tube resistance	η	108.0	g cm^{-1} h^{-1}	Minchin et al., 1993
	T	295.0	K	This study
	R	$1.078 \cdot 10^{15}$	g cm^2 h^{-2} mol^{-1} K^{-1}	Gas constant
Water density	d_p	0.9250	cm^3 H_2O cm^{-3} plant	This study

Soil volume	V_{soil}	0.135	ℓ	This study
Phosphate uptake	$U_{max}S_{max}$	33.297	μg h^{-1}	This study
	m_U	$1.479 \cdot 10^4$	μg l^{-1}	This study
	m_{as}	0.160	cm^3	This study
Respiration	c_l^{su}	0.035	μg sugar μg^{-1} sugar loaded	Cannell & Thornley, 2000
	c_e	0.053	μg sugar μg^{-1} phosphate taken up	Cannell & Thornley, 2000
