# Supplementary material for: Mathematical Modeling of the Dynamics of Shoot-Root Interactions and Resource Partitioning in Plant Growth
Source: PLoS One. 2015 Jul 8;10(7):e0127905. doi: 10.1371/journal.pone.0127905 (PMC4495989; doi:10.1371/journal.pone.0127905)
Supplement: S2 Table — (PDF) [file pone.0127905.s005.pdf]

Table S2. Fitted parameters of the mathematical model.

Category	Parameter	Units	Initial value	Biological interval	Fitted Value	Source
Growth	g_{max}^{sh}	$cm^3 h^{-1}$	0.203	(0.183, 0.223)	0.199	This study
	g_{max}^{r}	$cm^3 h^{-1}$	0.084	(0.076, 0.092)	0.088	This study
	$m_{g,sh}$	cm^3	19.349	(17.414, 21.284)	21.053	This study
	$m_{g,r}$	cm^3	7.983	(7.185, 8.782)	7.817	This study
	$a_{g,sh,su} = a_{g,r,su}$	$(\mu g\ cm^{-3})^2$	1.372	(1.234, 1.509)	1.366	This study
	$a_{g,sh,ph} = a_{g,r,ph}$	$(\mu g\ cm^{-3})^2$	$6.304 \cdot 10^4$	$(5.674 \cdot 10^4, 6.935 \cdot 10^4)$	$6.845 \cdot 10^4$	This study
	$C_{su,gr}^{sh} = C_{su,gr}^{r}$	$\mu g\ cm^{-3}$	360.415	(324.373, 396.456)	324.389	This study
	$C_{ph,gr}^{sh} = C_{su,gr}^{r}$	$\mu g\ cm^{-3}$	69.693	(62.724, 76.663)	76.038	This study
Photosynthesis	P_{max}	mol CO_2 $m^{-2} s^{-1}$	22.700	(20.000, 55.000)	52.230	Steven Adams (personal com.)

Category	Parameter	Units	Initial value	Biological interval	Fitted Value	Source
	α	μ mol CO_2 (J PAR)$^{-1}$	0.055	(0.044, 0.065)	0.049	Steven Adams (personal com.)
	$m_{F,ph}$	μ g cm^{-3}	100.000	(30.000, 350.000)	47.806	This study
	$s_{F,st}$	(μ g cm^{-3})^2	$2.250 \cdot 10^8$	$(2.250 \cdot 10^6, 2.250 \cdot 10^{10})$	$2.250 \cdot 10^8$	This study
	$sd_{F,st}$	dimensionless	0.010	(0.001, 0.200)	0.182	This study
	$s_{F,su}$	(μ g cm^{-3})^2	$4.225 \cdot 10^7$	$(4.225 \cdot 10^5, 4.225 \cdot 10^9)$	$4.227 \cdot 10^7$	This study
	$sd_{F,su}$	dimensionless	0.010	(0.0001, 1.000)	0.161	This study
Respiration	$m_{R,1}^{sh} = m_{R,1}^{r}$	μ g cm^{-3} h^{-1}	33.333	(10.000, 66.667)	48.273	Rasse & Tocquin (2006)
	$m_{R,2}^{sh} = m_{R,2}^{r}$	μ g cm^{-3} h^{-1} $(\mu g\ cm^{-3})^{-1}$	0.085	(0.026, 0.255)	0.254	Rasse & Tocquin (2006)
	$g_R^{sh} = g_R^{r} =: g_R$	μ g cm^{-3}	$8.070 \cdot 10^3$	$(7.263 \cdot 10^3, 8.877 \cdot 10^3)$	$8.548 \cdot 10^3$	Amthor (2000)

Category	Parameter	Units	Initial value	Biological interval	Fitted Value	Source
Phloem	r	cm	$5.0 \cdot 10^{-3}$	$(5.000 \cdot 10^{-4}, 5.000 \cdot 10^{-2})$	$5.536 \cdot 10^{-3}$	Sheehy *et al.*, 1995
Sugar anabolism	θ_{su}^{sh}	μ g cm $^{-3}$	$7.740 \cdot 10^{4}$	$(6.966 \cdot 10^{4}, 8.514 \cdot 10^{4})$	$7.047 \cdot 10^{4}$	Rains, 1976
	θ_{su}^{r}	μ g cm $^{-3}$	$7.954 \cdot 10^{4}$	$(7.158 \cdot 10^{4}, 8.749 \cdot 10^{4})$	$7.165 \cdot 10^{4}$	Rains, 1976
Transpiration	E_d	mol H_2O cm $^{-2}$ h $^{-1}$	$1.440 \cdot 10^{-4}$	$(1.296 \cdot 10^{-4}, 1.584 \cdot 10^{-4})$	$1.323 \cdot 10^{-4}$	Lambers *et al.*, 1998
	E_n	mol H_2O cm $^{-2}$ h $^{-1}$	$1.152 \cdot 10^{-4}$	$(1.037 \cdot 10^{-4}, 1.267 \cdot 10^{-4})$	$1.038 \cdot 10^{-4}$	Zeeman (personal com.): $0.8 \cdot E_d$
Phosphate transport	p_{max}^{r}	dimensionless	0.05	(0.010, 1.000)	0.061	This study
	p_{max}^{sh}	dimensionless	0.070	(0.010, 1.000)	0.052	This study
Target sugar concentration	$C_{su,t}^{sh}$	μ g cm $^{-3}$	$1.0 \cdot 10^{3}$	$(5.000 \cdot 10^{2}, 2.500 \cdot 10^{3})$	$8.693 \cdot 10^{2}$	This study

Category	Parameter	Units	Initial value	Biological interval	Fitted Value	Source
Starch	s_{st}	$(\mu g\ cm^{-3})^2$	$6.400 \cdot 10^{11}$	$(6.400 \cdot 10^9, 6.400 \cdot 10^{13})$	$6.400 \cdot 10^{11}$	This study
	sd_{st}	dimensionless	0.010	(0.001, 0.200)	0.142	This study
	$a_{\rho,1}$	$(\mu g\ cm^{-3})^2$	$2.250 \cdot 10^6$	$(2.250 \cdot 10^4, 2.250 \cdot 10^8)$	$2.276 \cdot 10^6$	This study
	$s_{\rho,2}$	$(\mu g\ cm^{-3})^2$	250.0	(2.500, 25000.000)	105.566	This study
	$s_{\rho,3}$	$(\mu g\ cm^{-3})^2$	350.0	$(3.500, 3.500 \cdot 10^4)$	$2.257 \cdot 10^4$	This study
	k_1	h^{-1}	0.005	(0.001, 0.015)	0.011	This study
	k_2	h^{-1}	0.010	(0.001, 0.030)	0.020	This study
	k_3	h^{-1}	0.020	(0.002, 0.060)	0.014	This study
