# Supplementary material for: Mathematical Modeling of the Dynamics of Shoot-Root Interactions and Resource Partitioning in Plant Growth
Source: PLoS One. 2015 Jul 8;10(7):e0127905. doi: 10.1371/journal.pone.0127905 (PMC4495989; doi:10.1371/journal.pone.0127905)
Supplement: S3 Table — (PDF) [file pone.0127905.s006.pdf]

Table S3 Parameter values of Thornley's model (resulting from a fit to our experimental data)

Parameter	Description	Units	Initial value	Fitted Value	Interval	Source
k_G^s	Shoot growth rate	(g su/g FW) $^{-1}$ (g Pi/g FW) $^{-1}$ d $^{-1}$	$2.321 \cdot 10^5$	$2.303 \cdot 10^5$	$(5.802 \cdot 10^4, 9.283 \cdot 10^5)$	This study
k_G^r	Root growth rate	(g su/g FW) $^{-1}$ (g Pi/g FW) $^{-1}$ d $^{-1}$	$2.771 \cdot 10^5$	$2.869 \cdot 10^5$	$(6.928 \cdot 10^4, 1.108 \cdot 10^6)$	This study
k_{lit}	Litter production parameter	d $^{-1}$	$8.557 \cdot 10^{-3}$	$8.597 \cdot 10^{-3}$	$(2.139 \cdot 10^{-3}, 3.423 \cdot 10^{-2})$	This study
K_{Mlit}	Litter production parameter	g	47.439	56.926	(11.860,189.754)	This study
$k_C\ S_{max}$	Photosynthesis rate	g su d $^{-1}$	1.0745	1.047	(0.269,4.298)	Steve Adams (Personal communications)
K_{Ms}		g	8.225	6.839	(2.056,32.902)	Arbitrary
K_{Mr}		g	0.192	0.205	$(4.800 \cdot 10^{-2}, 0.768)$	This study
J_C	Carbon production inhibition	g su (g FW) $^{-1}$	$4.0 \cdot 10^{-3}$	$8.676 \cdot 10^{-5}$	$(4.0 \cdot 10^{-6}, 4.0)$	Arbitrary
q	scaling parameter	dimensionless	1	1	1	Thornley (1998b)
ρ_C	Carbon transport resistance	(g FW) $^{q-1}$ d	0.05	0.0207	$(1.250 \cdot 10^{-2}, 0.200)$	Arbitrary
f_C	Anabolism	g su (g FW) $^{-1}$	$2.625 \cdot 10^{-2}$	$2.454 \cdot 10^{-2}$	$(6.563 \cdot 10^{-3}, 0.105)$	1.125 g $C_6H_{12}O_6$

						per g of dry weight
k_P	Uptake rate max	g d $^{-1}$	$9.495 \cdot 10^{-4}$	$8.579 \cdot 10^{-4}$	$(2.374 \cdot 10^{-4}, 3.798 \cdot 10^{-3})$	This study
J_P	Phosphate production inhibition	g Pi (g FW) $^{-1}$	$2.942 \cdot 10^{-2}$	0.429	$(2.942 \cdot 10^{-5}, 29.420)$	This study
ρ_P	Phosphate transport resistance	(g FW) $^{q-1}$ d	1	0.381	(0.25,4)	Thornley (1995)
f_P	Anabolism	g Pi (g FW) $^{-1}$	0	0	0	
k_{CL}		μmol photons m^{-2}s^{-1}	397.577	402.807	$(99.394, 1.590 \cdot 10^{3})$	Steve Adams (Personal communications)
k_{PR}		μ g Pi l $^{-1}$	$1.802 \cdot 10^{4}$	$1.955 \cdot 10^{4}$	$(4.505 \cdot 10^{3}, 7.208 \cdot 10^{4})$	This study
